# Lamellipodin promotes invasive 3D cancer cell migration via regulated interactions with Ena/VASP and SCAR/WAVE

**DOI:** 10.1038/onc.2016.47

**Published:** 2016-03-21

**Authors:** G Carmona, U Perera, C Gillett, A Naba, A-L Law, V P Sharma, J Wang, J Wyckoff, M Balsamo, F Mosis, M De Piano, J Monypenny, N Woodman, R E McConnell, G Mouneimne, M Van Hemelrijck, Y Cao, J Condeelis, R O Hynes, F B Gertler, M Krause

**Affiliations:** 1 Koch Institute for Integrative Cancer Research at MIT, Massachusetts Institute of Technology, Cambridge, MA, USA; 2 King's College London, Randall Division of Cell and Molecular Biophysics, London, UK; 3 King's College London, Research Oncology, Division of Cancer Studies, Faculty of Life Sciences and Medicine, London, UK; 4 Department of Anatomy and Structural Biology, Albert Einstein College of Medicine, Bronx, NY, USA; 5 Gruss Lipper Biophotonics Center, Albert Einstein College of Medicine, Bronx, NY, USA; 6 Department of Microbiology, Tumor and Cell Biology, Karolinska Institute, Stockholm, Sweden; 7 King's College London, Division of Cancer Studies, Cancer Epidemiology Group, London, UK; 8 King's College London, Division of Cancer Studies, Richard Dimbleby Department of Cancer Research, London, UK; 9 University of Arizona Cancer Center, Tucson, AZ, USA; 10 Howard Hughes Medical Institute, Massachusetts Institute of Technology, Cambridge, MA, USA

## Abstract

Cancer invasion is a hallmark of metastasis. The mesenchymal mode of cancer cell invasion is mediated by elongated membrane protrusions driven by the assembly of branched F-actin networks. How deregulation of actin regulators promotes cancer cell invasion is still enigmatic. We report that increased expression and membrane localization of the actin regulator Lamellipodin correlate with reduced metastasis-free survival and poor prognosis in breast cancer patients. In agreement, we find that Lamellipodin depletion reduced lung metastasis in an orthotopic mouse breast cancer model. Invasive 3D cancer cell migration as well as invadopodia formation and matrix degradation was impaired upon Lamellipodin depletion. Mechanistically, we show that Lamellipodin promotes invasive 3D cancer cell migration via both actin-elongating Ena/VASP proteins and the Scar/WAVE complex, which stimulates actin branching. In contrast, Lamellipodin interaction with Scar/WAVE but not with Ena/VASP is required for random 2D cell migration. We identified a phosphorylation-dependent mechanism that regulates selective recruitment of these effectors to Lamellipodin: Abl-mediated Lamellipodin phosphorylation promotes its association with both Scar/WAVE and Ena/VASP, whereas Src-dependent phosphorylation enhances binding to Scar/WAVE but not to Ena/VASP. Through these selective, regulated interactions Lamellipodin mediates directional sensing of epidermal growth factor (EGF) gradients and invasive 3D migration of breast cancer cells. Our findings imply that increased Lamellipodin levels enhance Ena/VASP and Scar/WAVE activities at the plasma membrane to promote 3D invasion and metastasis.

## Introduction

Breast cancer metastasis is one of the leading causes of cancer-associated mortality in women worldwide.^1^ Metastasis is a multistep process.^2^ After breaching, the basement membrane metastasizing cancer cells migrate through the dense extracellular matrix (ECM) of the tumor stroma in order to intravasate.^2, 3^ Carcinoma cells that migrate in a mesenchymal mode form elongated membrane protrusions driven by the assembly of branched F-actin networks. Actin polymerization-driven migration and invasion is coordinated by the proto-oncogenes c-Src and c-Abl kinases and cytoskeletal regulatory proteins including Rac GTPase, the Scar/WAVE complex and Ena/VASP proteins.^4, 5, 6, 7^

Ena/VASP proteins (Mena, EVL and VASP) enhance processive filament elongation.^8, 9, 10, 11, 12, 13, 14^ Mena is upregulated in breast cancer and promotes invasion.^15, 16^ We identified Lamellipodin (Lpd) as a binding partner of Ena/VASP proteins.^5, 17, 18^ Lpd localizes to lamellipodia, thin membrane protrusions at the leading edge of migrating cells.^17^ The Lpd-Ena/VASP interaction is positively regulated by Abl kinase-mediated Lpd phosphorylation, which drives Ena/VASP recruitment to lamellipodia by Lpd.^19^

Lpd is required for lamellipodium formation^17^ and binds directly to the Scar/WAVE complex.^20^ Scar/WAVE activates the Arp2/3 complex to nucleate branched actin networks during lamellipodia formation.^4, 5, 6, 7^ Surprisingly, Lpd-driven random cell migration in 2D requires Lpd binding to Scar/WAVE, but not to Ena/VASP.^20^

The mechanisms by which actin regulators coordinate the interplay between actin-elongation and actin-branching factors to promote cancer cell invasion remain incompletely understood. Here, we report that Lamellipodin mediates invasive 3D migration of cancer cells via selective, regulated interactions with Ena/VASP and Scar/WAVE. Our findings point to key roles for increased Lpd levels in breast cancer invasion and metastasis.

## Results

**Figure 1:**
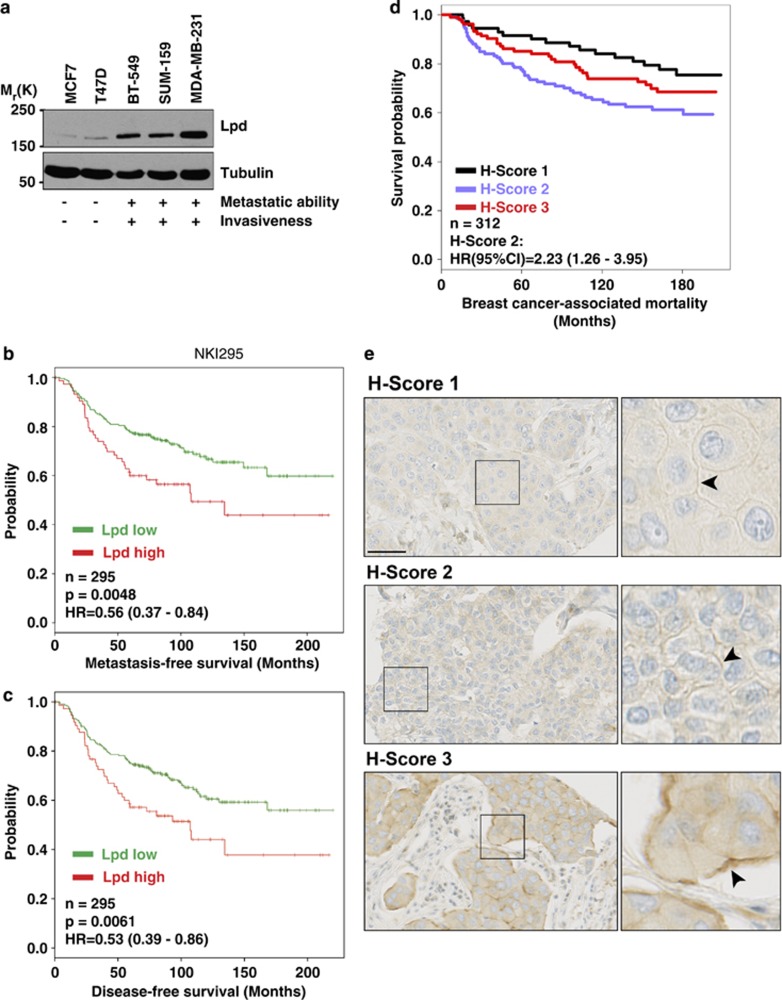
Increased Lpd expression correlates with poor prognosis for breast cancer patients. (**a**) Western blot analysis of Lpd expression in human breast cancer cell lines with varying metastatic potential. Loading control: Tubulin. (**b**) Kaplan–Meier analysis of metastasis-free survival in the NKI295 data set. Patients were stratified by expression of Lpd. The *P*-value was calculated by a log-rank test. (**c**) Kaplan–Meier analysis of disease-free survival in the NKI295 data set. Patients were stratified by expression of Lpd. The *P-*value was calculated by a log-rank test. (**d**) Kaplan–Meier plots of breast cancer-associated mortality of histoscores 1–3 for Lpd intensity at the plasma membrane. Histoscore 2: HR (95% CI): 2.23 (1.26–3.95). (**e**) Representative examples of Lpd immunohistochemistry staining for histoscores 1–3 for Lpd staining intensity at the plasma membrane. Scale bar, 5 μm. See also Supplementary Figures S1 and S2.

We observed higher Lpd levels in invasive and metastatic basal cell lines compared with noninvasive, luminal tumor cell lines (Figure 1a). Therefore, we analyzed publicly available data sets^21^ to examine whether Lpd mRNA levels correlated with occurrence of distant metastases in breast cancer patients. Lpd was overexpressed in several types of breast tumors compared with matched healthy tissue (Supplementary Figure 1A). High levels of Lpd mRNA correlated with reduced metastasis-free and disease-free survival of breast cancer patients in three separate cohorts (Figures 1b and c; Supplementary Figures 1B and C).^22, 23, 24^ In addition, we explored whether Lpd protein expression levels correlate with clinical outcome for breast cancer patients by staining a tumor microarray (TMA)^25^ generated from 312 patients with invasive breast cancer with anti-Lpd antibodies. Moderately, but not highly, increased abundance of Lpd in the cytoplasm (Histoscore 2; Hazard ratio (HR) (95% confidence interval (CI)): 1.765 (1.026–3.036); Supplementary Figures 1D and 2A,B) and at the plasma membrane (Histoscore 2: HR, (95% CI): 2.231 (1.26–3.949); Figures 1d and e; compared with respective histoscore 1) was significantly associated with increased risk for breast cancer-associated mortality. Furthermore, we observed an inverse correlation between Lpd intensity at the plasma membrane and Her2 expression (Supplementary Figure 1E). Consistent with Lpd's predominant role at the plasma membrane in promoting cell motility and migration,^17, 19, 20^ we observed a significant association between highly, but not moderately, increased Lpd staining intensity at the plasma membrane and reduced disease-free (Histoscore 3: HR (95% CI): 1.652 (1.24–2.428)) and metastasis-free survival of breast cancer patients (Histoscore 3: HR (95% CI): 1.515 (1.054–2.178); Figure 1e compared with respective histoscore 1).

**Figure 2:**
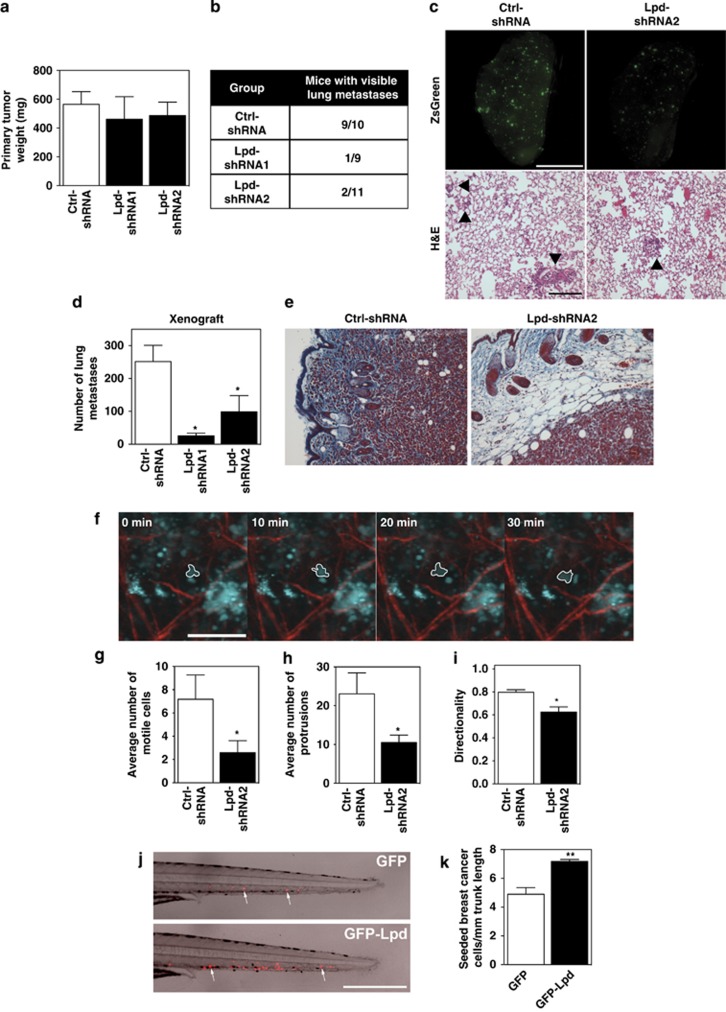
Lpd is required for lung metastasis from orthotopic mammary tumors. (**a–e**) NOD/SCID/IL2Rγ-null mice were injected orthotopically with LM2 cells stably expressing Ctrl-shRNA or Lpd-shRNA1 or Lpd-shRNA2. Tumors were allowed to grow for 6±0.5 weeks. (**a**) Primary tumor weights at the time of killing of individual mice are shown. Data are represented as mean±s.e.m. Number of animals per group is indicated in (**b**). (**b**) The number of mice that presented with visible metastases in the lung is indicated (mice with lung metastases/total number of mice analyzed). (**c**) Representative images of whole left pulmonary lobe from LM2 (control or knockdown) tumor-bearing mice with ZsGreen-positive metastatic foci (top panel). Scale bar, 5 mm. Representative lung sections stained with H&E; arrowhead indicates the presence of metastatic foci (bottom panel). Scale bar, 20 μm. (**d**) Numbers of ZsGreen-positive metastatic foci in the left pulmonary lobe were counted. Quantification of data shown in (**c**, top panel). Data are represented as mean±s.e.m. One-way ANOVA; Dunnett's; *P⩽0.05. Number of animals per group is indicated in (**b**). (**e**) Representative images of paraffin tissue sections stained with Masson's trichrome of primary tumors to show local invasion. Scale bar, 20 μm. (**f–i**) Tumor cell motility *in vivo* monitored by multi-photon confocal imaging. (**f**) Image shows a Ctrl-shRNA ZsGreen tumor. Cyan=ZsGreen-positive cells, red=collagen fibers. One motile Ctrl-shRNA-expressing tumor cell is outlined. Scale bar, 40 μm. (**g**) The average numbers of motile cells per field were determined. Data are represented as mean±s.e.m.. Unpaired *t*-test; *P⩽0.05, *n*=5 mice per group. (**h**) The average numbers of cells extending protrusions per field were determined. Data are represented as mean±s.e.m.; unpaired *t*-test; *P⩽0.05; *n*=5 mice per group. (**i**) Directionality of the motile cells (net path/total path) was determined. Data are represented as mean±s.e.m.; unpaired *t*-test; *P⩽0.05; *n*=5 mice per group. (**j, k**) Red fluorescently labeled MDA-MB-231 cells overexpressing GFP-Lpd or GFP as a control were implanted into the perivitelline cavity of zebrafish embryos and dissemination to the trunk region was quantified 2 days after injection. (**j**) Image of representative zebrafish trunks 2 days after injection. Arrows point to seeded tumor cells. Scale bar, 500 μm. (**k**) Quantified data from (**j**) represented as mean number of seeded tumor cells per mm of trunk length. Mean±s.e.m; data from 53 fish embryos for GFP and from 49 fish embryos for GFP-Lpd from 3 independent experiments; *t*-test; **P⩽0.01. See also Supplementary Figure S2.

To investigate the requirement for Lpd in metastasis, we tested the effect of reducing Lpd expression in MDA-MB-231-LM2 cells (further referred to as LM2), a highly metastatic derivative of MDA-MB-231 breast cancer cells,^26^ on their ability to metastasize from an orthotopic mammary tumor to the lungs. We generated stable LM2 cell lines with two independent Lpd-targeting shRNAs or a non-targeting control shRNA, all three retroviruses also conferring the cis-linked marker ZsGreen (Supplementary Figure 2C). Lpd knockdown and control cells were injected orthotopically into the mammary fat pad of immunodeficient mice. Primary tumors formed 6 weeks after injection from Lpd-deficient cells were similar in size to those arising from control LM2 cells, suggesting that the loss of Lpd did not affect primary tumor growth (Figure 2a). Importantly, only 3 out of 20 mice bearing Lpd-depleted tumors developed macroscopic lung metastases, compared with 9 out of 10 control tumor-bearing mice (Figure 2b). In addition, animals with tumors generated from Lpd-depleted cells that metastasized displayed significantly reduced numbers of pulmonary ZsGreen-positive metastases compared with the metastatic burden of animals with control tumors (Figures 2c and d).

We then explored whether Lpd promotes cancer cell invasion during metastasis. In fixed samples, tumors generated from control shRNA LM2 cells prominently invaded the surrounding stroma. However, tumors from Lpd knockdown cells were markedly less invasive (Figure 2e). We investigated Lpd function in cancer invasion in more detail by intravital imaging. Compared with control shRNA LM2 tumors, Lpd knockdown tumors had fewer motile cells, which migrated less directionally and extended protrusions less frequently (Figures 2f–i; Supplementary Video 1), indicating that Lpd is required for invasive cancer cell phenotypes *in vivo*.

To examine Lpd-driven breast cancer intravasation and dissemination, we implanted fluorescently labeled MDA-MB-231 breast cancer cells into the perivitelline cavities of zebrafish embryos. In this assay, the injected cancer cells intravasate, and then infiltrate the trunk of the fish.^27, 28^ Overexpression of GFP-Lpd in MDA-MB-231 cells enhanced the frequency of seeding of these breast cancer cells compared with GFP-expressing control cells (Figures 2j and k). We then performed tail vein injections of the Lpd knockdown and control LM2 cell lines into immunocompromised mice and quantified lung metastases after 28 days to test whether Lpd influenced the later stages of the metastatic cascade. Lpd depletion did not reduce the number of metastatic foci in the lungs of the mice compared with controls (Supplementary Figures 2D and E). Taken together, our results reveal that Lpd promotes local tumor invasion, intravasation and metastasis *in vivo,* but is not required for extravasation.

**Figure 3:**
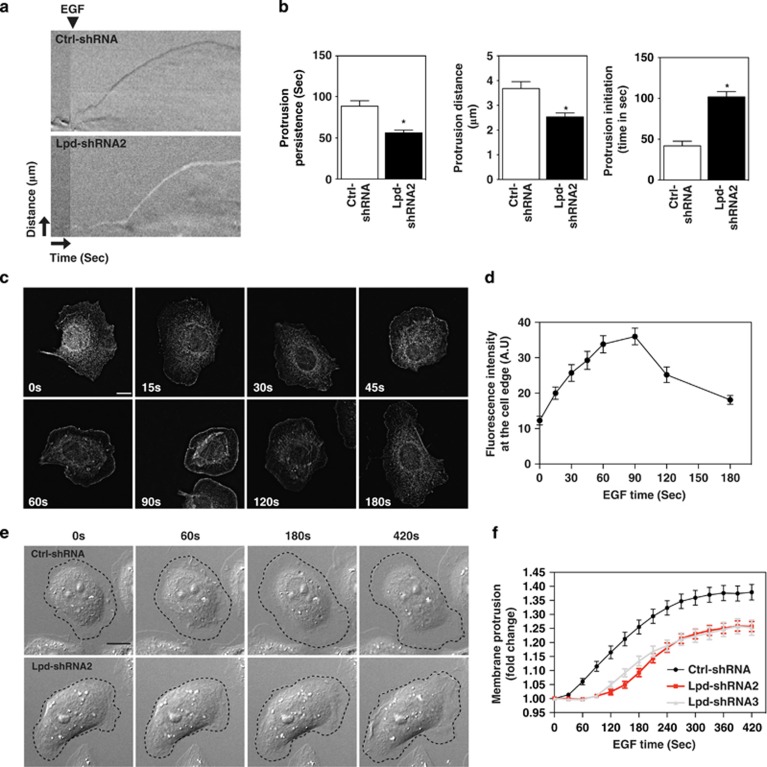
Lpd is required for EGF-induced membrane protrusion. (**a**) Representative kymographs of Ctrl-shRNA and Lpd-shRNA2 MTLn3 cells. A line drawn perpendicular to the cell surface is shown for each frame of a time-lapse movie to depict temporal dynamics of cell edge. X axis: time (arrow length: 20 s); Y axis: distance (arrow length: 3.1 μm). (**b**) Quantification of protrusion parameters from kymographic analysis of Ctrl-shRNA and Lpd-shRNA2 MTLn3 cells. Data represented as mean±s.e.m. Unpaired *t*-test; *P⩽0.05. (**c**) Micrographs showing immunofluorescence for endogenous Lpd in MTLn3 cells stimulated with 5 nm EGF (post-stimulation time is indicated). Scale bar, 10 μm. (**d**) Quantification of data shown in (**c**): mean fluorescence intensity of Lpd within a 0.66-μm zone at the lamellipodial edge is plotted as a function of time; >30 cells analyzed from at least three independent experiments. Error bars indicate s.e.m. (**e**) Representative micrographs from time-lapse movies of Ctrl-shRNA (control non-targeting shRNA) and Lpd-shRNA2-expressing MTLn3 cells stimulated with 5 nm EGF. Dashed line shows cell edge. Scale bar, 10 μm. (**f**) Quantification of membrane protrusions on Ctrl-shRNA and Lpd-shRNA-treated cells. Cell area was determined after EGF stimulation and normalized to the pretreatment cell area; >30 cells analyzed from three independent experiments. Error bars indicate s.e.m. See also Supplementary Figure S3.

Breast cancer cell migration toward blood vessels is guided by cues from the tumor microenvironment, such as epidermal growth factor (EGF).^29^ We reasoned that the effect of Lpd depletion on EGF-induced 3D invasion might arise from defects in lamellipodial dynamics. Depletion of Lpd in MDA-MB-231 breast cancer cells decreased lamellipodia size (Supplementary Figure 3A), similar to B16-F1 mouse melanoma cells, in which Lpd depletion also reduces protrusion speed under steady-state conditions.^17^ EGF-stimulated MDA-MB-231 Lpd knockdown cells displayed reduced protrusion persistence and distance, without affecting protrusion speed (Supplementary Figure 3B). We chose MTLn3 cells, a mammary adenocarcinoma cancer cell line, in which protrusion responses to EGF have been extensively characterized, to examine EGF-elicited protrusion in more detail as lamellipodial size is least affected by Lpd knockdown in this cell line (Supplementary Figure 3A). In agreement with our findings in MDA-MB-231 cells, in EGF-stimulated MTLn3 cells reduced Lpd levels significantly decreased protrusion persistence and distance (Figures 3a and b) but did not affect protrusion speed (Supplementary Figure 3D), compared with controls. Lpd was diffusely distributed throughout the cytoplasm of serum-starved cells, but was rapidly recruited to the cell edge following bath application of EGF (Figures 3c and d). Lamellipodial initiation was detected 30 s after EGF stimulation in Ctrl-shRNA-expressing cells but was delayed significantly when Lpd levels were reduced (Figures 3b, e and f; Supplementary Figure 3E; Supplementary Video 3). Taken together, our data suggest that, in breast cancer cells, Lpd depletion reduces EGF-elicited lamellipodial protrusion formation and persistence, but not speed.

**Figure 4:**
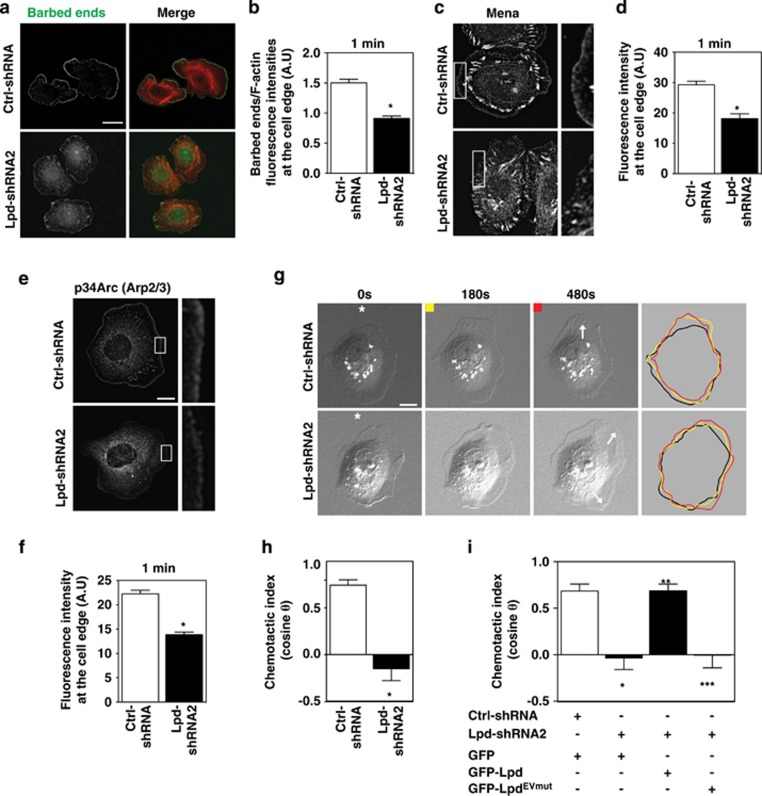
Lpd is required for chemosensing. (**a**) Barbed-end incorporation after 5 nM EGF stimulation in Ctrl-shRNA- and Lpd-shRNA2-MTLn3 cells. Fixed cells expressing rhodamine-labeled actin were co-stained with phalloidin. Scale bar, 20 μm. (**b**) Relative number of barbed-ends incorporation at the lamellipodium edge at 1 min after 5 nM EGF stimulation; over 60 cells analyzed. (*n*=3). Data are represented as mean±s.e.m. Unpaired *t*-test; *P⩽0.05. (**c**) Mena immunofluorescence in Ctrl-shRNA and Lpd-shRNA2 MTLn3 cells. Cells were stimulated for 1 min with 5 nM EGF. Insets show enlarged image of Mena staining. Scale bar, 10 μm. (**d**) Quantification of data shown in (**c**); mean fluorescence intensity of Mena at the lamellipodium edge (within 0.66 μm of leading edge); over 45 cells analyzed. (*n*=3). Data are represented as mean±s.e.m. Unpaired *t*-test; *P⩽0.05. (**e**) p34Arc immunofluorescence in Ctrl-shRNA- and Lpd-shRNA2-MTLn3 cells, 1 min after 5 nM EGF stimulation. Insets show enlarged image of p34Arc staining. Scale bar, 10 μm. (**f**) Quantification of data shown in (**e**); mean fluorescence intensity of p34Arc at the lamellipodium edge (within 0.66 μm of the leading edge); over 45 cells analyzed. (*n*=3). Data are represented as mean±s.e.m. Unpaired *t*-test; *P⩽0.05. (**g**) Representative micrographs from time-lapse movies of Ctrl-shRNA- and Lpd-shRNA2-MTLn3 cells stimulated with an EGF-filled micropipette (position indicated by asterisk). White arrows on the 480-s frames indicate the directions of protrusion overtime. Scale bar, 10 μm. Colored lines indicate cell contour. (**h**) Quantification of chemotactic index of Ctrl-shRNA- and Lpd-shRNA2-MTLn3 cells. Over 25 cells analyzed from at least three independent experiments. Data are represented as mean±s.e.m. Unpaired *t*-test; *P⩽0.05. (**i**) Quantification of chemotactic index of Ctrl-shRNA MTLn3 cells transfected with GFP-vector (*n*=13 cells); and Lpd-shRNA2 MTLn3 cells transfected with either GFP-vector (*n*=22 cells), GFP-Lpd (*n*=17 cells) or GFP-Lpd^EVmut^ (*n*=17 cells). Data are represented as mean±s.e.m. One-way ANOVA; Bonferroni's test; *P⩽0.05 vs Ctrl-shRNA+GFP; **P⩽0.05 vs Lpd-shRNA2+GFP; ***P⩽0.05 vs Lpd-shRNA2+GFP-Lpd. The difference between Lpd-shRNA2+GFP and Lpd-shRNA2+GFP-Lpd^EVmut^ was not significant. See also Supplementary Figures S4 and S5.

Membrane extension during lamellipodial protrusion is driven by actin polymerization.^4, 5, 6^ To determine how Lpd depletion influences actin polymerization, we used a G-actin incorporation assay^30^ to measure the abundance and distribution of polymerization-competent, free (uncapped) F-actin barbed-ends in lamellipodia of living cells. Silencing of Lpd significantly reduced free barbed-end formation 1 min after EGF stimulation, relative to Ctrl-shRNA-expressing cells (Figures 4a and b). Collectively, these data indicate that Lpd promotes lamellipodial protrusion by increasing actin polymerization downstream of EGFR activation.

EGF-dependent membrane protrusion in MTLn3 cells requires Ena/VASP proteins and Arp2/3-mediated dendritic nucleation;^16, 31^ and Lpd binds both Ena/VASP proteins and the Arp2/3 activating Scar/WAVE complex.^17, 19, 20^ Consistent with this, membrane recruitment of Mena (Figures 4c and d) and Arp2/3 complex to the protruding edge (Figures 4e and f) was significantly reduced in Lpd-depleted cells after EGF stimulation.

We hypothesized that the requirement for Lpd in EGF-induced protrusion reflects Lpd-mediated initiation of chemotactic responses. The initial step of chemotaxis is directional sensing.^32, 33^ We used a micropipette to generate a spatially restricted EGF gradient. Ctrl-shRNA MTLn3 cells formed new protrusions toward the pipette, demonstrating their ability to sense the EGF gradient. However, those of Lpd-reduced cells were random relative to the micropipette (Figures 4g and h; Supplementary Figures 4A and B; Supplementary Video 5). Chemotactic indices confirmed the lack of directional bias in Lpd-deficient MTLn3 protrusions (Figure 4h), highlighting an essential role for Lpd in the initial steps of chemotaxis toward EGF.

Lpd is concentrated at the edges of lamellipodia that protrude in response to uniform EGF stimulation. Correlative differential interference contrast microscopy and immunofluorescence imaging in live cells revealed enrichment of Lpd at the edges of cells oriented toward the micropipettes containing EGF (Supplementary Figure 4C), confirming that Lpd was enriched in membranes exposed to the highest concentration of the EGF gradient and supporting a function for Lpd in linking gradient sensing to directed membrane protrusion.

We hypothesized that EGF chemosensing might involve Lpd-mediated recruitment of Ena/VASP proteins. We tested whether Lpd-depleted cells, expressing either a GFP-Lpd or an Lpd mutant in which all Ena/VASP binding sites had been rendered non-functional by mutation (GFP-Lpd^EVmut^), could respond to EGF gradients in the micropipette assay and discovered that, while GFP-Lpd effectively rescued the chemosensing defects in Lpd-depleted cells, GFP-Lpd^EVmut^ conferred no significant phenotypic rescue (Figure 4i and Supplementary Figure 5C). In line with this finding, a function-perturbing approach^34^ revealed that Ena/VASP proteins were required for chemosensing (Supplementary Figures 5A and B). Thus, extension of lamellipodia toward EGF during chemosensing by breast carcinoma cells requires the Lpd-dependent recruitment of Ena/VASP proteins, despite the dispensability of Ena/VASP for Lpd-driven random 2D cell migration.

**Figure 5:**
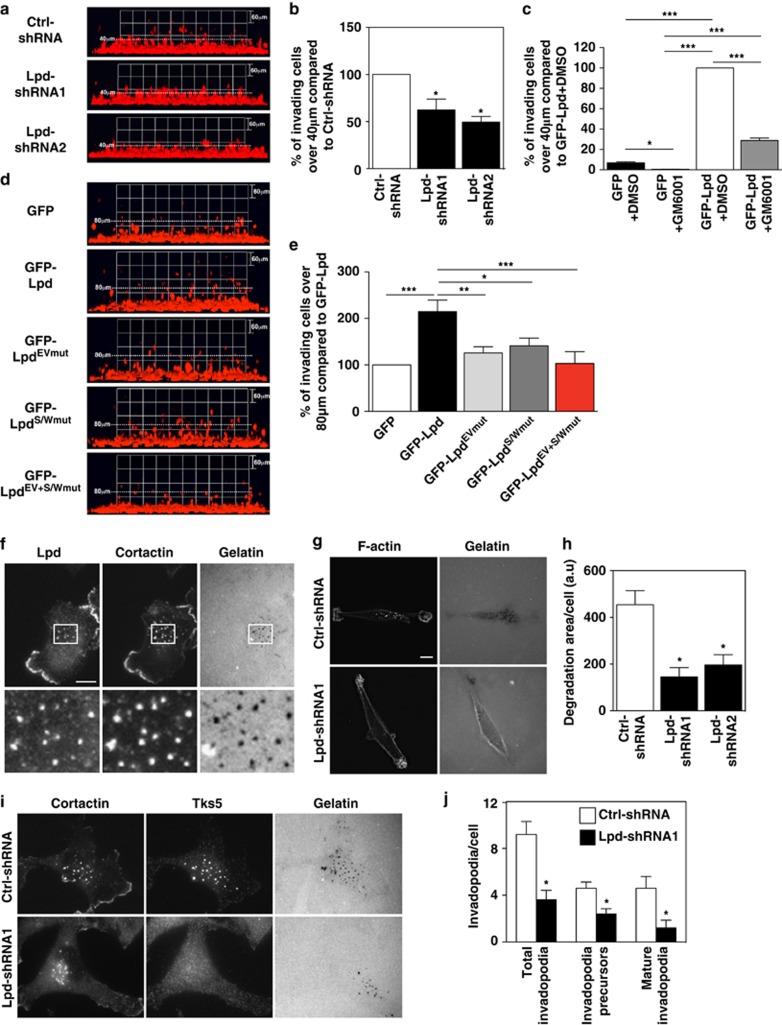
Lpd is required for 3D invasion of cancer cells. (**a, b**) Inverted invasion assays were performed using MDA-MB-231 cells stably expressing mCherry-H2B (labeling the nucleus) transfected with Ctrl-shRNA, Lpd-shRNA1 or Lpd-shRNA2. Additionally, cells were co-transfected with empty Blasticidin vector as well and transfected cells were selected. The nuclei of the cells were visualized using confocal microscopy. (**a**) The image stacks were processed by Volocity software to make a 3D reconstruction. (**b**) Quantification of the number of nuclei of invading cells above 40 μm from the data shown in (**a**), *n*=4 (with approximately 4000 cells per experiment). Data are represented as mean±s.e.m. One-way ANOVA; Dunnett's; *P⩽0.05. (**c**) Inverted invasion assays with GFP or GFP-Lpd expressing MDA-MB-231 cells treated with the MMP inhibitor 10 μM GM6001 or just the solvent DMSO as a control. Quantification of the number of nuclei of invading cells above 40 μm from the data shown in Supplementary Figure 6E. *n*=3. Data are represented as mean±s.e.m. One-way ANOVA; Tukey's; *P⩽0.05, **P⩽0.01, ***P⩽0.001. (**d**) Inverted invasion assays were performed using MDA-MB-231 breast cancer cells stably expressing mCherry-H2B (labeling the nucleus) transfected with GFP-Lpd, GFP-Lpd^EVmut^, GFP-Lpd^S/Wmut^, GFP-Lpd^EV+S/Wmut^ or GFP empty vector as a control. The nuclei of the cells were visualized using confocal microscopy. The image stacks were processed by Volocity software to make a 3D reconstruction. (**e**) Quantification of the number of nuclei of invading cells above 40 μm from the data shown in (**d**), *n*=3 (with approximately 4000 cells per experiment). Data are represented as mean±s.e.m. One-way ANOVA; Dunnett's; *P⩽0.05, **P⩽0.01, ***P⩽0.001. (**f**) MDA-MB-231 plated on Alexa 488-gelatin/fibronectin matrix, fixed and stained for Lpd, and the invadopodia marker cortactin. White boxes: enlarged images shown in insets. Scale bar: 10 μm. (**g**) Steady-state assay for invadopodial matrix degradation. MDA-MB-231 cells were plated for 8 h on Alexa 488-gelatin/fibronectin matrices, fixed and stained with phalloidin. Scale bar, 10 μm. (**h**) Quantification of data shown in (**g**): invadopodial degradation area/cells in the steady-state matrix degradation assay, normalized to number of cells/field. Data are represented as mean±s.e.m. (*n*=3). Mann–Whitney test; *P<0.05. (**i**) MDA-MB-231 stably expressing Ctrl-shRNA or Lpd-shRNA cells were plated on 405-gelatin and immunostained with cortactin and Tks5 antibodies to identify invadopodia. Scale bar, 10 μm. (**j**) Quantification of data shown in (**i**); number of total invadopodia, invadopodia precursors and mature invadopodia per cell were determined; Ctrl-shRNA cells (*n*=52) or Lpd-shRNA cells (*n*=57). Data are represented as mean±s.e.m. Mann–Whitney test; *P<0.05. See also Supplementary Figure S6.

We next analyzed the requirements for Lpd during EGF-dependent 3D invasion. In 3D inverted chemotaxis assays toward EGF with MDA-MB-231 or SUM-159 invasive breast cancer lines^35, 36^ knockdown of Lpd significantly decreased invasion through matrigel (Figures 5a and b; Supplementary Figure 6A) and collagen (Supplementary Figures 6B–D) compared with control shRNA expressing cells. Conversely, Lpd overexpression significantly increased invasion toward EGF (Figures 5d and e).

As invasion is known to be partially dependent on matrix metalloproteinase (MMP) digestion of ECM, we tested whether Lpd increases invasion via MMP-dependent or migration-dependent mechanisms. MMP inhibitor treatment of GFP-expressing control cells reduced invasion, as expected. Similarly, MMP inhibitor treatment reduced invasion of Lpd overexpressing MDA-MB-231 cells. However, Lpd overexpressing cells treated with MMP inhibitors invaded significantly further compared with GFP control cells treated with the inhibitor, suggesting that Lpd functions to increase invasion by increasing migration and potentially MMP-dependent ECM degradation (Figure 5c and Supplementary Figure 6E).

The aforementioned findings prompted us to investigate Lpd's role in MMP-dependent degradation. Carcinoma cells can utilize protrusive invadopodia, sites of MMP exocytosis, to invade through the basement membrane and ECM-rich interstitial stroma.^37, 38, 39^ We observed that Lpd co-localized with the invadopodial marker cortactin at invadopodia, at sites of matrix degradation (Figure 5f). Previously, we reported that Mena promotes invadopodium stabilization and matrix degradation,^16^ and here we observed that Lpd depletion appeared to reduce the amount of Mena within invadopodia considerably, potentially reflecting a role for Lpd in Mena recruitment to invadopodia (Supplementary Figure 6H). Silencing of Lpd in MDA-MB-231 cells decreased the number of precursors, mature invadopodia and the total number of invadopodia in comparison with control cells (Figures 5i and j). Furthermore, Lpd-depleted MDA-MB-231 cells exhibited a significant decrease in their ability to degrade matrix relative to control cells (Figures 5g and h). Taken together, these results suggest that Lpd is required for invadopodial precursor formation or for both precursor formation and subsequent stabilization/maturation, and for invadopodia-mediated matrix degradation.

To test the relative contribution of Lpd interactions with Ena/VASP proteins or with the Scar/WAVE complex during MDA-MB-231 3D invasion, we overexpressed a panel of Lpd mutants in which all Ena/VASP (GFP-Lpd^EVmut^), all Scar/WAVE-binding sites (GFP-Lpd^S/Wmut^), or all Ena/VASP and all Scar/WAVE-binding sites (GFP-Lpd^EV+S/Wmut^) had been mutated. All of these mutants localized to the leading edge of MDA-MB-231 cells (Supplementary Figures 6F and G). At steady state when embedded in 3D matrigel, MDA-MB-231 cells overexpressing GFP-Lpd displayed significantly more protrusions, which protruded faster compared with GFP control cells. However, expression of Lpd-Ena/VASP-, Lpd-Scar/WAVE- and double-binding mutants all failed to increase protrusion numbers and speed (Figure 7h and Supplementary Figure 7G). Similarly, these mutants did not support invasion through matrigel toward EGF, suggesting that Lpd promotes 3D chemotactic invasion via both Ena/VASP and Scar/WAVE (Figures 5d and e). These findings, combined with our previous observation that Lpd interaction with Scar/WAVE but not with Ena/VASP is required for random 2D migration, prompted us to hypothesize that interactions between Lpd and these actin regulators may be differentially regulated.

Previously, we found that Lpd is phosphorylated by Abl kinases upon growth factor stimulation, and this positively regulates its interaction with Ena/VASP proteins and their recruitment to the leading edge of cells.^19^ As Src kinases are also activated upon growth factor stimulation and increased Src activity promotes cancer cell invasion,^40^ we explored whether Lpd interactions with downstream partners are regulated by Src phosphorylation.

**Figure 6:**
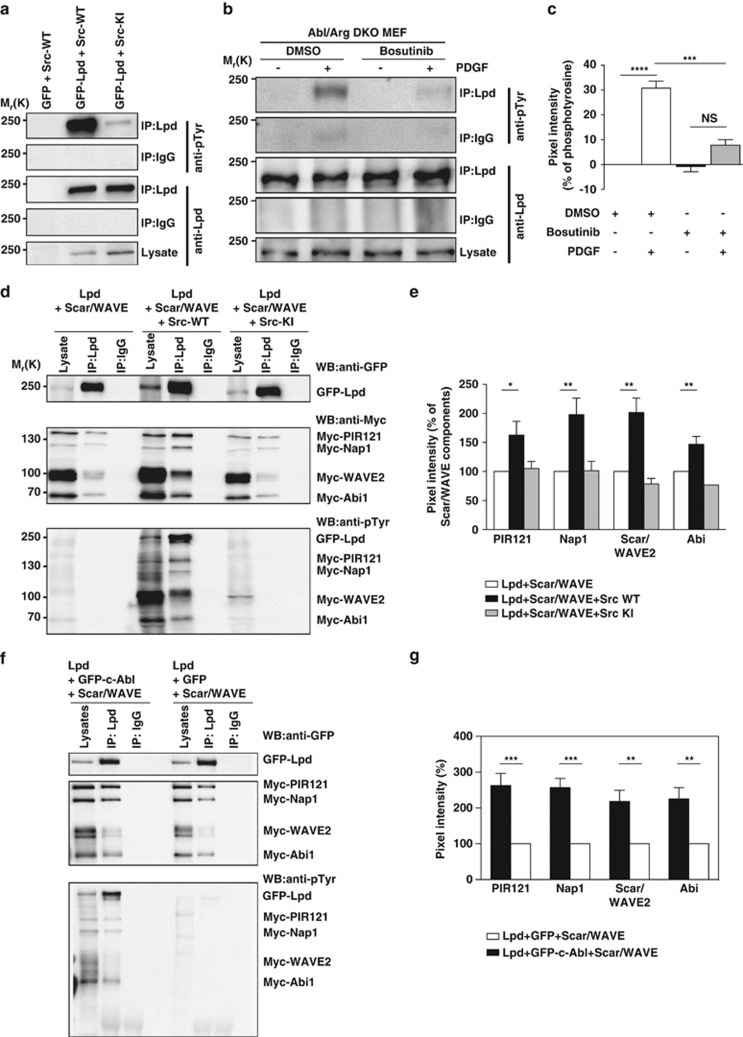
c-Src phosphorylates Lpd and the Lpd-Scar/WAVE interaction is positively regulated by c-Abl and c-Src. (**a**) HEK293FT cells were transfected with either GFP as a control or GFP-Lpd and co-transfected with Src-WT (wild type) or Src-KI (kinase inactive). Immunoprecipitation was performed from cell lysates using Lpd-specific antibodies or rabbit IgG as a control followed by western blotting with anti-Lpd and anti-phosphotyrosine (pTyr) antibodies. *n*=3. (**b**) Abl and Arg double knockout MEFs (Abl/Arg DKO) were serum starved overnight and treated with 10 μm Bosutinib (c-Src kinase inhibitor) for 2 h before stimulating with 20 ng/ml PDGF-BB for 2 min. Immunoprecipitation was performed from cell lysates using Lpd-specific antibodies or rabbit IgG as a control followed by western blotting with anti-Lpd and anti-phosphotyrosine (pTyr) antibodies. (**c**) Quantified band intensities of chemiluminescence blots from (**b**) of Lpd and pTyr imaged with a CCD camera. pTyr was normalized against the immunoprecipitated Lpd. The pTyr signal from Rabbit IgG control lanes was subtracted from the pTyr signal from the immunoprecipitated Lpd lanes. *n*=3, data are represented as mean±s.e.m. One-way ANOVA; Dunnett's; ***P⩽0.001, ****P⩽0.0001, NS, not significant. (**d**) HEK293FT cells were transfected with GFP-Lpd, Myc-tagged components of the Scar/WAVE complex and either Src-WT (wild type) or Src-KI (kinase inactive). Immunoprecipitation was performed from cell lysates using Lpd-specific antibody or rabbit IgG as a control followed by western blotting with anti-GFP, anti-Myc and anti-phosphotyrosine (pTyr) antibodies. (**e**) Quantified band intensities of chemiluminescence blots (**d**) of GFP-Lpd and Myc-tagged components of the Scar/WAVE complex imaged with a CCD camera. Individual Scar/WAVE components were normalized against the immunoprecipitated Lpd. *n*=4, data are represented as mean±s.e.m. One-way ANOVA; Dunnett's; *P⩽0.05, **P⩽0.01. (**f**) HEK293FT cells were transfected with GFP-Lpd, Myc-tagged components of the Scar/WAVE complex and either GFP-c-Abl or GFP. Immunoprecipitation was performed from cell lysates using Lpd-specific antibody or rabbit IgG as a control followed by western blotting with anti-GFP, anti-Myc and anti-phosphotyrosine (pTyr) antibodies. (**g**) Quantified band intensities of chemiluminescence blots (**f**) of GFP-Lpd and Myc-tagged components of the Scar/WAVE complex imaged with a CCD camera. Individual Scar/WAVE components were normalized against the immunoprecipitated Lpd. *n*=4, data are represented as mean±s.e.m. One-way ANOVA; Dunnett's; **P⩽0.01, ***P⩽0.001.

We expressed GFP-Lpd with wild-type c-Src or a kinase-inactive mutant of c-Src and, after immunoprecipitation of Lpd, observed that it was tyrosine phosphorylated in cells expressing wild-type but not kinase-inactive c-Src (Figure 6a). Both Src and Abl kinases are activated downstream of growth factor receptors, and Src phosphorylation of Abl kinases contributes to their activation.^40, 41, 42^ To distinguish between Src and Abl phosphorylation of Lpd and to investigate whether endogenous Lpd is phosphorylated by Src tyrosine kinases, we made use of Abl/Arg double knockout mouse embryonic fibroblasts.^43^ After PDGF-BB stimulation, Lpd was robustly tyrosine phosphorylated in the absence of both Abl kinases, Abl and Arg. This was blocked by the Src inhibitor Bosutinib (Figures 6b and c), indicating that Src kinase activity leads to Lpd phosphorylation upon growth factor receptor activation.

We first investigated whether Src phosphorylation controls Lpd-Ena/VASP interaction, since this is positively regulated by Abl phosphorylation.^19^ Surprisingly, c-Src-induced Lpd phosphorylation did not affect Lpd-Ena/VASP binding (Supplementary Figures 7A and B). In contrast, co-immunoprecipitation between GFP-Lpd and Myc-tagged Scar/WAVE complex revealed that significantly more Scar/WAVE complex co-precipitated with Lpd when either c-Src or c-Abl was co-expressed (Figures 6d–g). We also tested whether the interaction between endogenous Lpd and Scar/WAVE is positively regulated by c-Src by using ectopically expressed GST-tagged Abi (which reduces endogenous Abi and thereby replaces it^44^) to efficiently pull down the Scar/WAVE complex and associated proteins. GST-Abi pulldowns from cells co-expressing GFP-Src contained higher levels of endogenous Scar/WAVE2 co-precipitating with endogenous Lpd compared with GFP controls (Supplementary Figures 7C and D). Conversely, GST-Abi pulldowns from cells co-expressing GFP-Src treated with the c-Src inhibitor (KB SRC 4) contained lower levels of endogenous Scar/WAVE2 co-precipitating with endogenous Lpd compared with GST-Abi pulldowns from DMSO-treated control cells (Supplementary Figures 7E and F). Together, these findings indicate that the Lpd-Ena/VASP interaction is regulated by c-Abl phosphorylation, whereas the Lpd-Scar/WAVE interaction is positively regulated by both c-Abl and c-Src-dependent phosphorylation.

**Figure 7:**
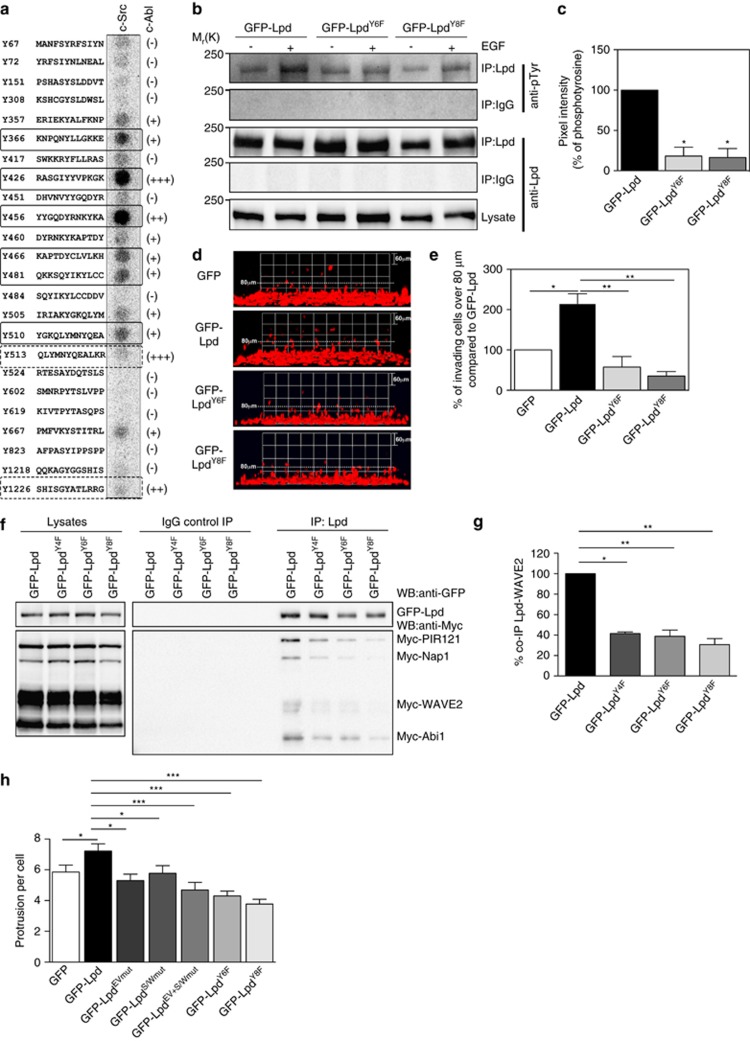
Phosphorylation of Lpd by c-Src and c-Abl is required for cancer cell invasion. (**a**) Peptides harboring all the tyrosine residues in Lpd were directly synthesized onto a membrane. An *in vitro* kinase assay was performed: the membranes were incubated with purified c-Src kinase and γ-^32^P-ATP. Phosphorylation was detected using a phosphorimager and visualized as high intensity spots. Increasing levels of c-Abl phosphorylation of respective peptides as identified by Michael *et al.*^19^ are indicated by (+), (++), (+++) and (−) for not phosphorylated. Straight rectangles represent the common phosphorylation sites for both c-Src and c-Abl, and dotted rectangles represent c-Abl specific phosphorylation sites.^19^ (**b**) HeLa cells were transfected with GFP-Lpd^Y6F^, GFP-Lpd^Y8F^ or GFP-Lpd as a control. HeLa cells were serum starved overnight and stimulated with 100 ng/ml EGF for 5 min. Immunoprecipitation was performed from cell lysates using Lpd-specific antibodies or rabbit IgG as a control followed by western blotting with anti-Lpd and anti-phosphotyrosine (pTyr) antibodies. (**c**) Quantified band intensities of chemiluminescence blots (**b**) of GFP-Lpd, GFP-Lpd phospho-mutants and pTyr imaged with a CCD camera. pTyr normalized against the immunoprecipitated Lpd. Baseline phosphorylation in the absence of EGF was subtracted from the corresponding EGF+ samples. *n*=6, data are represented as mean±s.e.m. One-way ANOVA; Dunnett's; *P⩽0.0001. (**d, e**) Inverted invasion assays were performed using MDA-MB-231 breast cancer cells stably expressing mCherry-H2B (labeling the nucleus) transfected with GFP-Lpd, GFP-Lpd^Y6F^, GFP-Lpd^Y8F^ or GFP empty vector as a control. The nuclei of the cells were visualized using confocal microscopy. (**d**) The image stacks were processed by Volocity software to make a 3D reconstruction. (**e**) Quantification of the number of nuclei of invading cells above 80 μm. *n*=6 (with approximately 4000 cells per experiment). Data are represented as mean±s.e.m. One-way ANOVA; Dunnett's; *P⩽0.001, **P⩽0.0001. Error bars represent s.e.m. (**f**) HEK293FT cells were transfected with GFP-Lpd, GFP-Lpd^Y4F^, GFP-Lpd^Y6F^, GFP-Lpd^Y8F^ and Myc-tagged components of the Scar/WAVE complex. Immunoprecipitation was performed from cell lysates using GFP-specific antibody or rabbit IgG as a control followed by western blotting with anti-GFP, anti-Myc and anti-phosphotyrosine (pTyr) antibodies. (**g**) Quantified band intensities of chemiluminescence blots (**f**) of GFP-Lpd and Myc-tagged components of the Scar/WAVE complex imaged with a CCD camera. Scar/WAVE2 was normalized against the immunoprecipitated Lpd. *n*=4, data are represented as mean±s.e.m. One-way ANOVA; Dunnett's; **P⩽0.01, ***P⩽0.001. (**h**) Quantification of the number of protrusion of MDA-MB-231 transfected with GFP-Lpd, GFP-Lpd^EVmut^, GFP-Lpd^S/Wmut^, GFP-Lpd^EV+S/Wmut^, GFP-Lpd^Y6F^, GFP-Lpd^Y8F^ or GFP empty vector as a control plated in 3D matrigel. *n*=35–46 cells for each mutant; from 5 experiments. Data are represented as mean±s.e.m. One-way ANOVA; Dunnett's; *P⩽0.05, ***P<0.001. See also Supplementary Figures S7 and S8.

To better understand this differential regulation, we identified potential direct c-Src phosphorylation sites in Lpd, using purified c-Src kinase to phosphorylate an immobilized peptide array covering all putative tyrosine phosphorylation sites in Lpd. This analysis revealed that Lpd harbors two robustly and four weakly phosphorylated c-Src tyrosine phosphorylation sites (Figure 7a). We have previously mapped eight c-Abl tyrosine phosphorylation sites in Lpd,^19^ which partly overlap with these newly identified c-Src sites (Figure 7a). To verify that the Lpd phosphorylation sites identified *in vitro* were phosphorylated in cells, we mutated the tyrosines in the six c-Src (GFP-Lpd^Y6F^) and the eight c-Abl phosphorylation sites (GFP-Lpd^Y8F^) to phenylalanine, rendering them non-phosphorylatable. Overexpression of GFP-Lpd induced low levels of Lpd tyrosine phosphorylation, which was markedly enhanced by stimulation with 100 ng/ml EGF for 5 min. In contrast, neither GFP-Lpd^Y6F^ nor GFP-Lpd^Y8F^ tyrosine phosphorylation was enhanced when cells expressing these constructs were stimulated with EGF (Figures 7b and c). We found that the GFP-Lpd^Y6F^ and GFP-Lpd^Y8F^ mutants interacted significantly less with the Scar/WAVE complex (Figures 7f and g; Supplementary Figure 8D). Nevertheless, GFP-Lpd^Y6F^ and -Lpd^Y8F^ mutants localized to the leading edge of MDA-MB-231 cells similar to wild-type Lpd (Supplementary Figures 6F and G). Taken together, our data indicate that full-length Lpd can be phosphorylated at these sites in cells upon EGFR activation, likely as a consequence of activated Abl and Src kinases.

To verify that both Src and Abl kinases are required for breast cancer invasion toward EGF, we tested the invasiveness of MDA-MB-231 cells with and without incubation with the Src and Abl inhibitors Dasatinib or STI571. As expected, we found that breast cancer cell invasiveness is impaired when Src or Abl kinases were inhibited (Supplementary Figures 8A and B).

To investigate the functional significance of Lpd tyrosine phosphorylation for breast cancer invasion, we compared the effects of overexpressing the non-phosphorylatable mutants, GFP-Lpd^Y6F^ or GFP-Lpd^Y8F^ with control GFP-Lpd. We observed that, in contrast to GFP-Lpd, neither of the mutants increased protrusion numbers or speed in cells embedded in matrigel at steady state, or the invasiveness of breast cancer cells through matrigel toward EGF (Figures 7d, e and h and Supplementary Figure 8C), suggesting that phosphorylation by both Abl and Src kinases is required for Lpd-mediated breast cancer invasion.

## Discussion

Here, we reveal that Lpd is required for metastasis in an orthotopic breast cancer mouse model, and that increased Lpd levels correlate with reduced metastasis-free survival in breast cancer patients. Lpd promotes metastasis *in vivo* by supporting tumor invasion and intravasation. Lpd function in metastasis may be mediated via both Ena/VASP and the Scar/WAVE complex since we observed that Lpd mediates breast cancer invasion via both actin effectors. Both Ena/VASP and Scar/WAVE are implicated in breast cancer metastasis by multiple lines of evidence.^15, 16, 45, 46^ Our results suggest that the pro-metastatic function of Lpd may, in part, involve coordinating the activities of these two distinct types of actin regulators to optimize chemotactic invasion and matrix degradation by invading tumor cells.

Here, we provide evidence that Lpd is a substrate of Src kinases and that phosphorylation of Lpd by Src positively regulates the Lpd-Scar/WAVE complex interaction, but not the Lpd-Ena/VASP interaction, whereas c-Abl-mediated phosphorylation of Lpd positively regulates both Lpd-Ena/VASP and Lpd-Scar/WAVE interaction. This differential regulation of Lpd-Ena/VASP or Lpd-Scar/WAVE recruitment may allow Lpd to fine-tune actin cytoskeletal dynamics via Ena/VASP-mediated actin filament elongation and Scar/WAVE-Arp2/3-mediated nucleation/branching, consistent with the context-dependent differential requirements for these interactions we observed. Lpd-driven random 2D cell migration requires Scar/WAVE, but not Ena/VASP,^20^ Lpd-dependent chemosensing in 2D requires Ena/VASP, and interactions with both SCAR/WAVE and Ena/VASP are required for 3D chemotaxis and migration. These findings lead to the interesting possibility that Lpd balances actin nucleation/branching and filament elongation activities to optimize protrusion morphology and dynamics during cellular responses to growth factors and ECM composition and organization.

Surprisingly, we observed that only moderately, but not highly increased levels of Lpd correlate with increased risk of breast cancer-associated mortality, suggesting that not all of Lpd functions (cell invasion, cell proliferation and EGFR endocytosis)^47, 48^ induced by high but not medium levels of Lpd may be beneficial for tumors cells. However, in agreement with its role in metastasis, we found that highly increased Lpd abundance at the plasma membrane of cancer cells in breast tumors correlates with reduced disease- and metastasis-free interval. The increased membrane accumulation of Lpd protein observed in our TMA analysis may reflect the known role of Lpd in regulating membrane protrusion in migrating cells.^17, 20^ Furthermore, consistent with the TMA analysis, we found that increased Lpd mRNA levels correlate with reduced metastasis-free survival of breast cancer patients.

On the basis of our findings, we propose that Lpd functions as an essential component of a pro-metastatic signaling pathway composed of Src and Abl kinases, Lpd, Ena/VASP and Scar/WAVE that promotes metastatic progression.

## Materials and methods

### Plasmids and shRNAs

GFP-VASP, pMSCV-mRFP1-FP4/AP4-mito,^34^ Myc-Pir121, -Nap1, -Abi1d, -WAVE2 in pRK5-Myc-DEST^20^ and Lpd^17^ in pENTR (Invitrogen) mutated using Quikchange (Agilent), transferred into pCAG-DEST-EGFP (Gateway) (pCAG-EGFP; C Cepko, Addgene,11150) and pCB6-Src-WT-EGFP, pCB6-Src-KI (kinase-inactive)-EGFP^49^ (M Way).

GFP-Lpd^S/Wmut^: ^20^(AAS82582.1) Site 1: (aa 968–978) GKKP(P>A) (P>A) T(P>A)Q(R>A)N; Site 2: (aa 1119–1129) APP(P>A)TR(P>A)K(R>A)ND; Site 3: (aa 1230–1244) RRGP(P>A)A(P>A)(P>A)(K>A)(R>A)DQNT.

GFP-Lpd^EVmut^: ^20^(AAS82582.1) FP4-1: aa 869 F>A; FP4-2: aa 916 F>A; FP4-3: aa927 F>A; FP4-4: aa 1064 F>A; FP4-5: aa1073 F>A; FP4-6: aa1082 L>A; FP4-7: aa1202 L>A.

GFP-Lpd^Y4F^: ^19^(AAS82582.1) Y>F: aa 426, 456, 513, 1226; GFP-Lpd^Y6F^: (AAS82582.1) Y>F: aa 366, 426, 456, 466, 481, 510; GFP-Lpd^Y8F^: (AAS82582.1) Y>F: aa 366, 426, 456, 466, 481, 510, 513, 1226.

pLJM1-H2BK-mCherry: histone H2BK amplified from A431 cell by RT–PCR cloned into pLJM1-mCherry (D Sabatini; Addgene plasmid #19319).

shRNAs were cloned into pLL3.7-Puro, miR30-MLS EGFP/mCherry^50^ or MSCV-ZsG-2A-Puro-miR30 retroviral vector.^51^ Cells were FACS sorted or puromycin (Invitrogen) selected and knockdown tested by western blotting. shRNA (5′–3′) used:

Human Lpd-shRNA1: TTTCCCCAAAAGATAATTCTG, humanLpd-shRNA2: TTCCCATACTTTGCAATGCGG, ratLpd-shRNA2: TAGAGCTCACAGTACTTTGGG, ratLpd-shRNA3: AAGAGGTCCAATCATAAGCTG and Control-shRNA (targeting luciferase): TTAATTAAAGACTTCAAGCGG (Figures 2, 3, 4 and Supplementary Figures S2–S6). Human Lpd-shRNA-1:GCGTCAAATCACAGAAACG, Human Lpd-shRNA-2:GCTCTGAATCAGGGAGAGA and Control-shRNA (Lpd-scrambled): GCCGATAACCGAGAATACC (Figures 2 and 5; Supplementary Figures S2 and S6).

### Cell culture and transfection

HEK293, BT549, MCF7 and T47D cells obtained from ATCC and maintained according to ATCC's protocol. SUM-159 and MTLn3 cells cultured as described.^52, 53^ HEK cells expressing GST-Abi2 and GFP-Src were treated for 1 h with 100 μm KB SRC 4 (Tocris) before lysis. Abl1/Abl2 double knockout MEFs (gift of T Koleske, Yale), MDA-MB-231 cells (gift of A Ridley) and MDA-MB-231 LM2 cells^26^ (gift from J Massague, MSKCC) were cultured in high-glucose DMEM, penicillin, streptomycin, 10% FBS. MTLn3 and MDA-MB-231 transfection was done using Lipofectamine 2000 (Invitrogen). Abl1/2DKO MEFs were serum starved (18 h) and treated with 10 μm Bosutinib (Cambridge Biosciences) (2 h) in DMSO or DMSO (control), then stimulated with 20 ng/ml PDGF-BB (2 min).

### Antibodies

Lpd pab 3917,^17^ Mena mab A351F7D9,^54^ Wave2 (Cell Signaling Technology), p34Arc (Millipore, 07-227), Tubulin (DM1A), Hsc70 mab (Santa Cruz), GFP mab (Roche), Myc mab (Sigma, 9E10), pTyr mab (Millipore, 4G10) and Vimentin (550513, Biosciences). Alexa-conjugated secondary antibodies, phalloidin (Invitrogen, Biotium) diluted 1:50–1000.

### Immunoprecipitation, GST-pulldowns and western blotting

Immunoprecipitation, GST-pulldowns and western blotting were performed as described.^20^

### Peptide array assay

Peptide array assay was done using Src kinase (NEB) as described.^19^

### Immunofluorescence microscopy

Cells were plated on glass coverslips, fixed and stained as described.^19^ Imaged using Deltavision microscope (Applied Precision, Olympus IX71, × 60/1.4NA, Softworx software) (SGI, Mountain View, CA, USA). Olympus IX-81 microscope (Metamorph, Photometrics CascadeII 512B camera, × 40 UPlanFL, × 60 PlanApoNA1.45 or × 100 UPlanApoS NA1.4 objectives) was used. Leading edge localization (Supplementary Figure 6G) was quantified by two blinded observers from independent data sets (*n*=32–38 cells for each mutant).

### Inverted invasion assay

In all, 5 × 10^5^ MDA-MB-231 cells stably expressing mCherry-H2B were seeded onto underside of 8 μm pore-size transwell inserts (Greiner Bio-One Ltd) containing matrigel (BD Biosciences, UK; polymerized 30 min (37 °C)). Inserts were inverted after cells adhered (4 h), placed in serum-free medium, and normal growth medium containing EGF (25 ng/ml) placed on top. Inhibitors used were GM6001 μm; STI571 10 μm and Dasatinib 10 nm. Seventy-two hours later, cells that did not cross the transwell filter were removed; invading cells visualized by confocal microscopy; 2.5 μm sections. The number of nuclei of invading cells above 40 or 80 μm was automatically quantified using the Volocity software.

### Membrane protrusion assays

Membrane protrusion assay for EGF-treated cells was performed as described.^16^ Kymographic analysis was performed to analyze the protrusion parameters including: protrusion persistence, distance, velocity and protrusion initiation after EGF stimulation.

For membrane protrusion assay in 3D matrigel, MDA-MB-231 cells were stained with CellTracker Green dye (ThermoFisher, UK) embedded in matrigel (BD Biosciences) in μ-slide chambers (81506; Ibidi, Germany). Four hours after plating, 5 min movies, one frame every 15 s; × 40; Olympus IX-81 were generated and protrusive activity around the entire circumference between frames automatically quantified from thresholded movies using ImageJ plugin ADAPT.^55^

### Micropipette assay

The micropipette assay was performed as described.^53, 56^

### Barbed-end assay

G-actin was extracted from rabbit muscle acetone powder and standard techniques used to gel-filter over a Superdex-200 gel filtration column. The G-actin was polymerized to F-actin in F-actin buffer (1 mm ATP, 5 mm MgCl_2_, 50 mm KCl, 50 mm Tris/HCl, pH 8.0), labeled with Rhodamine-X succinimidyl ester (Invitrogen; following manufacturer's instructions), depolymerized in G-actin buffer (0.2 mm ATP, 0.5 mm DTT, 0.2 mm CaCl_2_, 2 mm Tris/HCl, pH 8.0) to G-actin, and passed through PD-10 columns (GE Healthcare) to eliminate free rhodamine. The barbed-end assay in MTLn3 cells was performed as described.^16^ Images taken with deconvolution microscope; the ratio of the barbed-end intensity to phalloidin intensity along the edge (the zone between 0 and 0.66 μm inside the cell) quantified as described above.

### Invadopodium degradation and immunofluorescence

MDA-MB-231 cells used in this study were cultured on FN/gelatin matrix (for 8 h) or thin gelatin matrix (for 4 h), and treated as described.^57^

### Zebrafish tumor cell dissemination assay

The zebrafish tumor cell dissemination assay was done as described.^28^

### Mouse models

Tumor growth and spontaneous metastasis formation assayed by injecting tumor cells orthotopically into inguinal mammary fat pads (6- to 8-week-old female NOD/SCID/IL2Rγ-null mice) (Jackson Laboratory, Bar Harbor, ME, USA). Mice anesthetized with isoflurane, injected with 1.5 × 10^5^ cells in Hank's Balanced Salt Solution (Gibco); killed 6±0.5 weeks post injection; tumors dissected, weighed, flash frozen, stored (−80 °C) or fixed: 3.8% formaldehyde, imaged with a fluorescence microscope, and embedded in paraffin and sectioned. Lungs were collected, fixed: 3.8% formaldehyde, embedded in paraffin, sectioned and stained with hematoxylin and eosin (H&E). ZsGreen-positive foci were counted in left pulmonary lobe using ImageJ.

### Intravital imaging

Female NOD/SCID/IL2Rγ-null mice (6–8 weeks old) injected in the mammary fat pad with Ctrl-shRNA or LpdhsRNA2-expressing LM2 cells. Experiments were performed as described previously.^58^ Five mice per group were used. Collagen I fibers were imaged by second harmonic generated polarized light. Cell motility observed by time-lapse imaging: 30 min in 2-min cycles. Three-dimensional time-lapse videos were analyzed using Image J. Tumor cell motility was quantified manually. A cell was scored as motile if the translocation of the cell body was visible over the course of a 30-min video within a visual field that is defined in three dimensions as 50 μm by 512 × 512 pixels. Protrusion was defined as tumors cells showing a dynamic lamellipodia-like morphology. A protrusion was defined to be at least 5 μm long, but less than half the length of the cell. Directionality was calculated as described.^58^

### Statistics

Statistical analysis performed by ANOVA with appropriate *post hoc* tests (see figure legends), or Student's *t*-test using Prism 5 (GraphPAD Software). *P*-values <0.05 considered as significant.

### Clinical data sets analysis

Oncomine (www.oncomine.org) used for Lpd mRNA expression from microarray data.^21^ Statistical survival analysis (Kaplan–Meier plots) was done with ROCK (http://www.rock.icr.ac.uk). Expression value of Lpd was upper quartile (25% against rest) for the NKI295^24^ and Loi^22^ data sets or upper tertile (33% against rest) for the Miller^23^ data set. The log-rank *P*-value was used for statistical significance.

### Tissue microarrays

TMAs were performed with 0.6 mm^2^ cores of formalin-fixed, paraffin-embedded invasive breast tumor samples (312 consecutive patients) with clinicopathological data (King's Health Partners Cancer Biobank, London, UK). Automated immunohistochemistry (VENTANA Discovery UTLRA) of 3 μm TMA sections was performed, deparaffinized (EZ prep; Ventana Medical) (30 min, 72 °C), antigen retrieval in automated slide stainer (ULTRA CC2 solution, Ventana Medical) (68 min, 91 °C). Affinity purified polyclonal rabbit Lpd antibody in PBS (1:250) (32 min, room temperature) was used. Slides counterstained with hematoxylin II and bluing reagent (Ventana Medical) (4 min each), dehydrated: 1 × 70% IMS, 1 min, 2 × 100% IMS, 1.5 min, 3 × xylene, 1 min, mounted (Eukitt) and imaged (Leica microscope, × 40).

Intensity of Lpd in cytoplasm and membrane assessed on TMAs using weighted histoscore (H-Score) method: intensity in majority of cells assessed as negative and weak (1), or moderate and strong (2), then multiplied by the percentage of cells within this category. The weighted histoscore: 0–200 divided into thirtiles: Cytoplasma: histoscore 1 (0–88.75), histoscore 2 (88.75–170) and histoscore 3 (170–200); Membrane: histoscore 1 (0–25), histoscore 2 (25–95) and histoscore 3 (95–200). TMAs were assessed by two independent observers (UP and CG). Intensity scores that varied by more than a factor of one or a proportion by more than 20% were jointly reassessed and consensus reached. For all other cases, the mean score was used. Disease-specific survival curves generated using the Kaplan–Meier method. The log-rank test was used to compare statistically significant differences between subgroups. Univariate and multivariate analyses Cox proportional hazards regression models used to evaluate overall and breast cancer-specific death by histoscore of Lpd intensity in cytoplasm and at membrane. All analyses were performed using Statistical Analysis Systems (SAS) 9.4 (SAS Institute, Cary, NC, USA).

### Study approval

The study approval was obtained for TMA from NHS Research Ethics Committee, King's Health Partners Cancer Biobank, London, UK. Mice: all animal experiments and husbandry were approved by MIT's Department of Comparative Medicine and Committee on Animal Care. The study approval was obtained for Zebrafish from Northern Stockholm Experimental Animal Ethical Committee.
