# Supplementary material for: Lamellipodin promotes invasive 3D cancer cell migration via regulated interactions with Ena/VASP and SCAR/WAVE
Source: Oncogene. 2016 Mar 21;35(39):5155–69. doi: 10.1038/onc.2016.47 (PMC5031503; doi:10.1038/onc.2016.47)
Supplement: Supplementary Information [file onc201647x1.pdf]

Supplementary information

Supplementary Material and Methods

Inverted Invasion Assay on collagen

Cells were suspended in serum-free collagen I (2.3 mg/ml) supplemented with fibronectin (25 μg/ml) to a final concentration of 2 × 103 cells/100 μl. Aliquots (100 μl) were dispensed into 96-well Costar plates coated with 3% heat-inactivated bovine serum albumin. Plates were centrifuged at 300×g and incubated at 37°C/5% CO2 for 30 min, and EGF was added; cells were fixed after 24 hours in 4% paraformaldehyde in PBS and stained with 5 μg/ml Hoechst 33258 (Molecular Probes-Invitrogen). Samples were run in duplicate, and analyzed on a confocal microscope by taking optical z sections every 5 μm, starting at the bottom of the well. Two random microscopic fields were counted for each replicate. Nuclear staining was analyzed with the Imaris Cell and Imaris MeasurementPro of Imaris 6 software (Bitplane Scientific Software), and 3D reconstructions of invaded cells were made using the Spot component of this module. The invasion index (number of cells >30 μm divided by the total number of cells) was calculated.

Extravasation metastasis assay

For the experimental lung metastasis assay, 50000 cells in 100 μl of Hank's Balanced Salt Solution were injected into the lateral tail vein of 6- to 8-week-old female NOD/SCID/IL2Rγ-null mice. The mice were sacrificed 28 days post-injection and lungs were inflated with 3.8% formaldehyde imaged with a fluorescence microscope and subsequently fixed overnight with 3.8% formaldehyde. ZsGreen-positive foci were counted in the left pulmonary lobe using ImageJ and counts were manually curated as needed.

Supplemental Figure 1

A

Tumor type and comparison tissue	Fold change	p value	Reference
Ductal Breast Carcinoma in Situ vs. Normal	2.844	0.022	Radvanyi
Invasive Breast Carcinoma Stroma vs. Normal	5.256	1.60E-10	Finak
Invasive Ductal Breast Carcinoma vs. Normal	1.53	3.20E-15	TCGA
Invasive Breast Carcinoma vs. Normal	1.469	4.29E-09	TCGA
Mucinous Breast Carcinoma vs. Normal	1.74	0.02	TCGA
Mixed Lobular and Ductal Breast Carcinoma vs. Normal	1.373	0.006	TCGA
Invasive Lobular Breast Carcinoma vs. Normal	1.301	2.85E-04	TCGA
Invasive Ductal Breast Carcinoma Epithelia vs. Normal	1.229	0.036	Ma

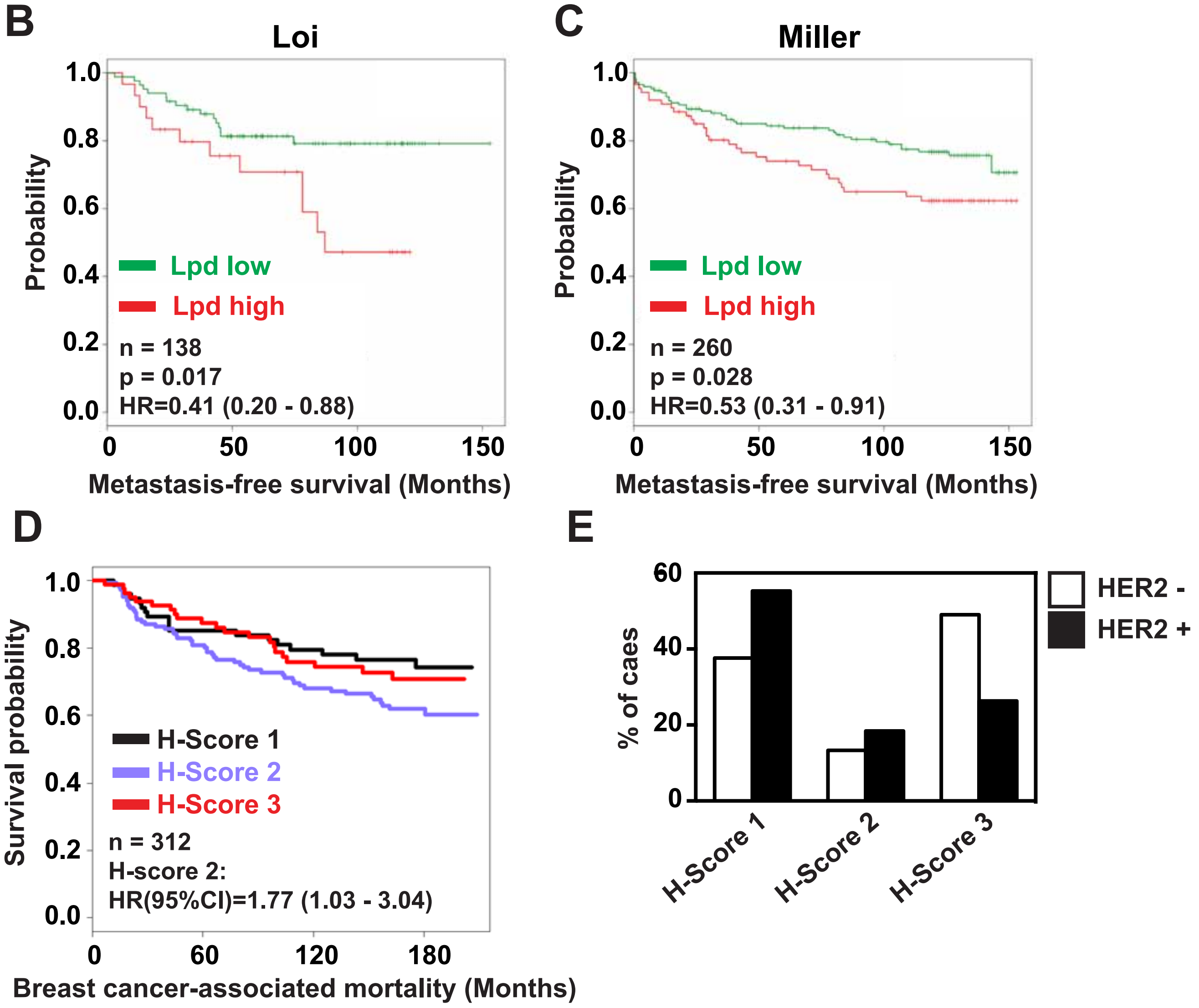

Supplemental Figure 1: Increased Lpd expression correlates with poor prognosis for breast cancer patients.
(A) Lpd expression was examined in various microarray data sets that profiled one or more breast tumor sub-types against normal tissue by using oncomine database. (B-D) Kaplan-Meier analysis of metastasis survival in the (B) Loi, (C) Miller datasets. Patients were stratified by expression of Lpd. The P value was calculated by a log rank test. (D) Kaplan Meier plots of breast cancer associated mortality of histoscore 1-3 for Lpd intensity in the cytoplasm. Histoscore 2: HR (95% CI): 1.765 (1.026-3.036). (E) Table showing the association between HER2 status and the histoscore 1-3 for Lpd intensity at the membrane. Chi-square = 6.7236; DF 2; $P \leq 0.0347$.

Supplemental Figure 2

A

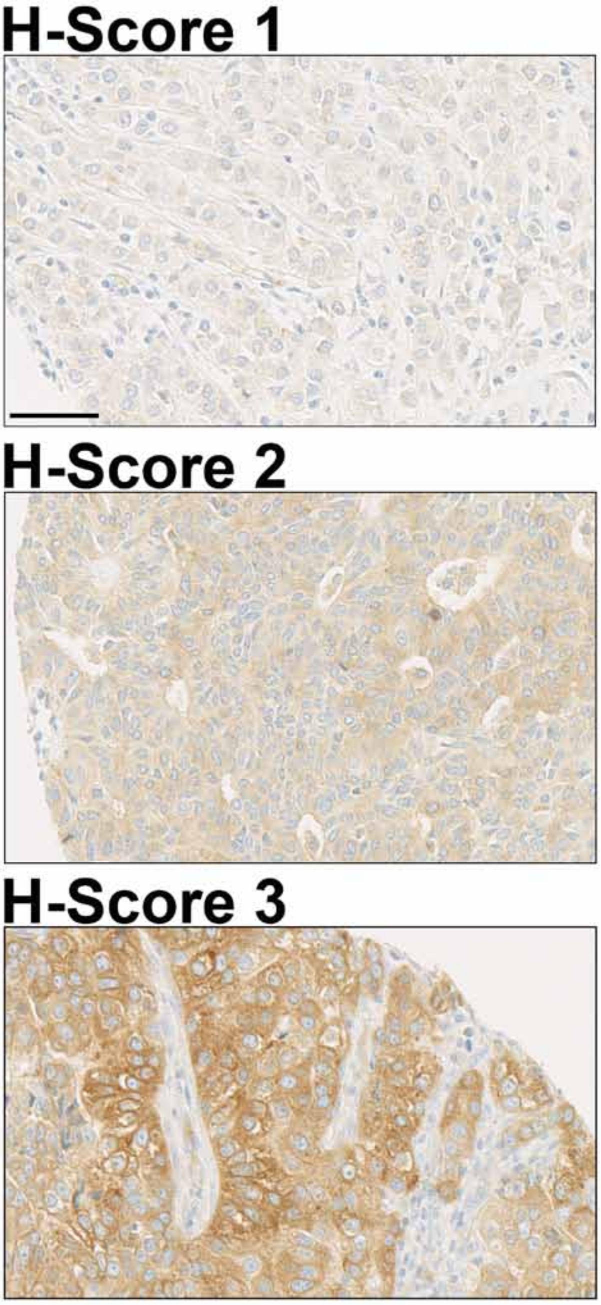

B

Clinical and pathological features of 312 primary breast carcinoma samples.

**Total Number**	312	
**Clinical Tumor Size[a] (cm)**		
Range	0.4 – 7.5	
Mean	2.53	
<2	42	15%
2-5	225	80%
>5	13	5%
**Histological Tumor Type**		
Ductal NOS[b]	257	82%
Lobular	37	12%
Pure Special Type	14	5%
Mixed/Other	4	1%
**Histological Grade**		
Grade I	52	17%
Grade II	122	39%
Grade III	113	36%
Unknown	25	8%
**Positive Axillary Lymph Nodes[c]**		
0	131	45%
1-3	97	33%
4+	63	22%
**Biomarkers[d]**		
ER	223	73%
PR	187	61%
HER2	248	79%
**Survival Status**		
Alive	152	
Died – Breast Cancer Cause	92	
Died – Other Causes	68	

[a]clinical size available for 280 cases.
[b]DuctalNOS = Ductal not otherwise specified.
[c]Nodal status available for 291 cases.
[d]Biomarker available for 306 cases.

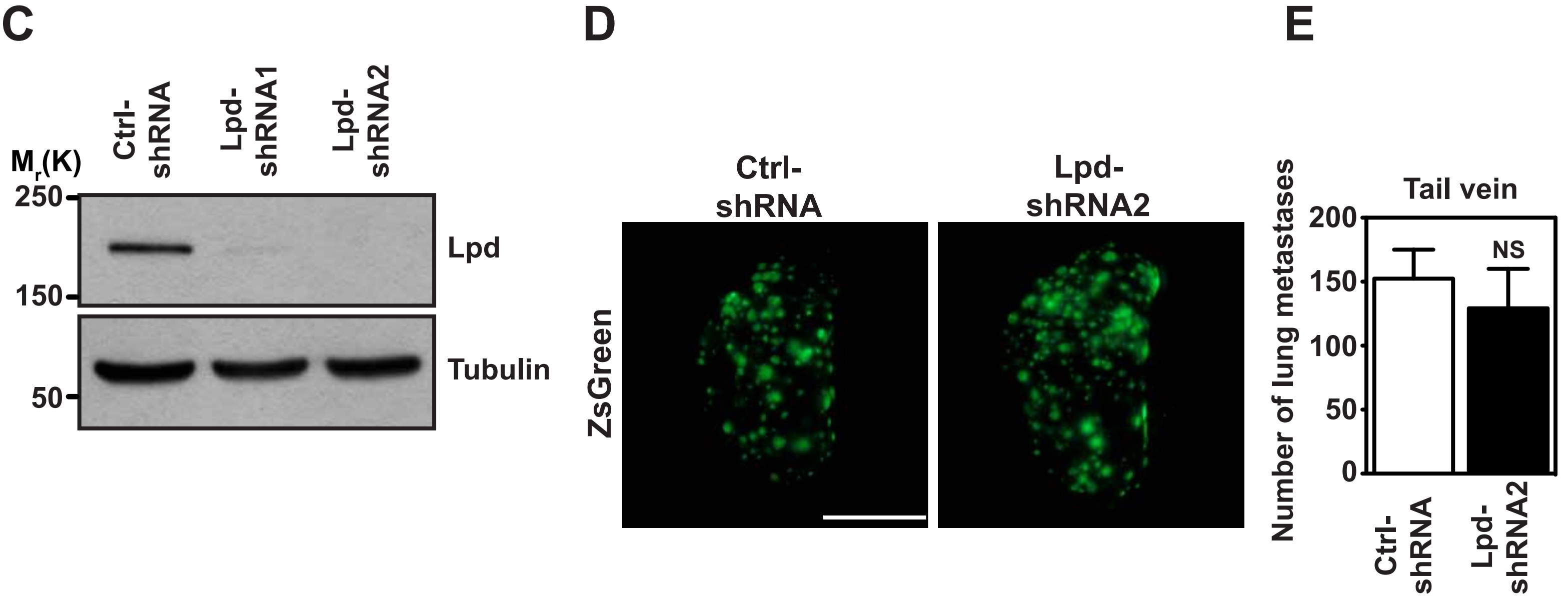

Supplemental Figure 2: Analysis of Lpd expression in tumor microarrays.

(A) Representative examples of Lpd immunohistochemistry staining for histoscore 1-3 for Lpd intensity in the cytoplasm. Scale bar, 5 µm. (B) Clinical and pathological features of the primary breast carcinoma samples. 312 patients analysed. (C) shRNA-mediated stable knockdown of Lpd in LM2 cells was measured by Western blotting. (D) LM2 cells stably expressing Ctrl-shRNA or Lpd-shRNA2 were injected via the tail vein and the formation of lung metastases was evaluated. Representative images of whole left pulmonary lobe from LM2 from mice injected with LM2 cells (Ctrl-shRNA or Lpd-shRNA2) with ZsGreen-positive metastatic foci (top panel). Scale bar, 5 mm. (E) LM2 cells stably expressing Ctrl-shRNA or Lpd-shRNA2 were injected via the tail vein and the formation of lung metastases was evaluated. Representative images of whole left pulmonary lobe from LM2 from mice injected with LM2 cells (Ctrl-shRNA or Lpd-shRNA2) with ZsGreen-positive metastatic foci (top panel). Scale bar, 5 mm. LM2 cells stably expressing Ctrl-shRNA or Lpd-shRNA2 were injected via the tail vein and, after 28 days, the formation of lung metastases was evaluated. Numbers of ZsGreen-positive metastatic foci in the left pulmonary lobe were counted. Data are represented as mean ± s.e.m. Number of animals per group: Ctrl-shRNA: 9 mice, Lpd-shRNA2: 9 mice. Unpaired t-test. The difference between and Ctrl-shRNA and Lpd-shRNA2 was not significant.

Supplemental Figure 3

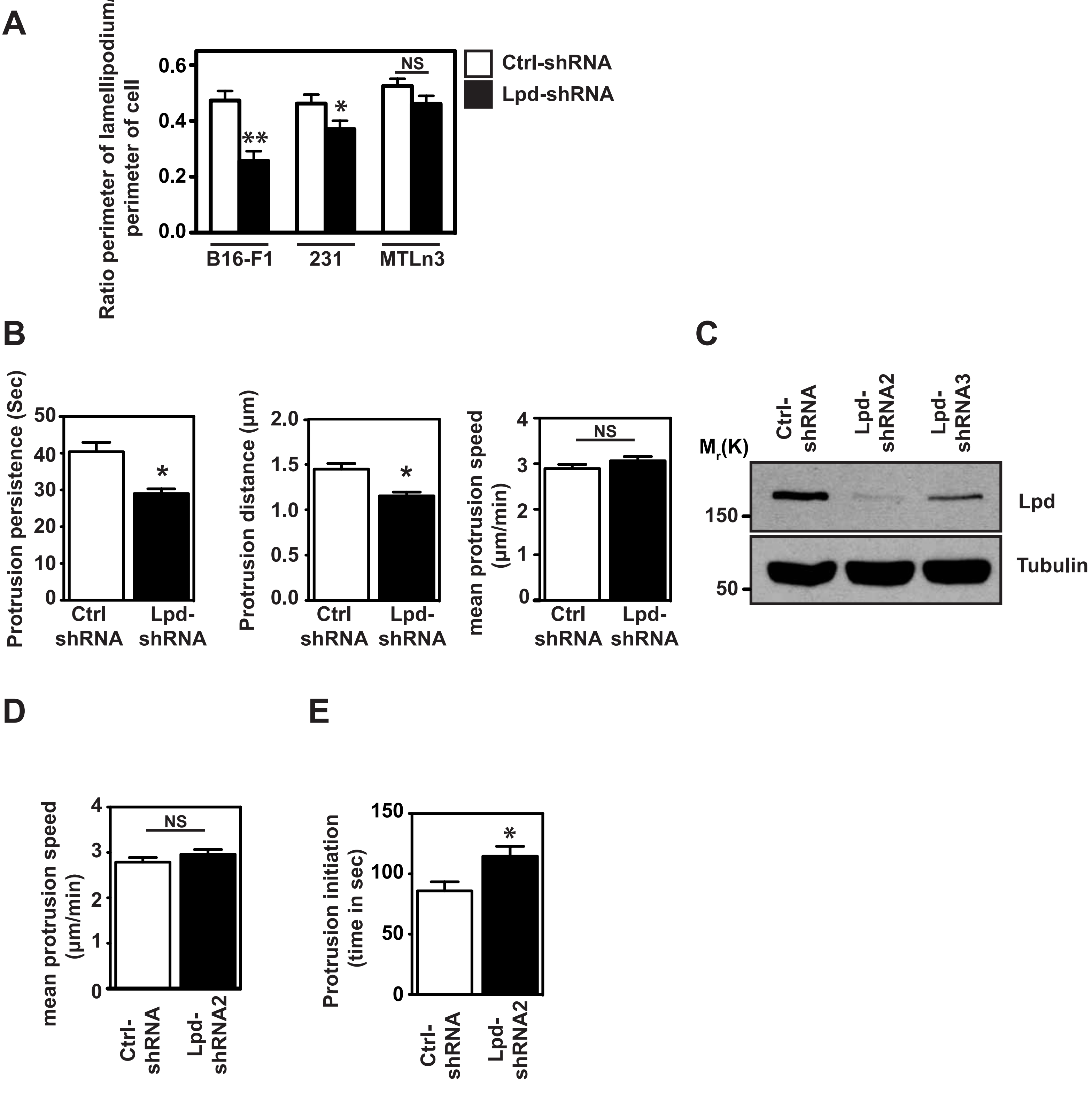

Supplemental Figure 3: Lpd is required EGF-induced membrane-protrusion.
(A) B16-F1, MDA-MB-231 and MTLn3 cell lines stably expressing Ctrl-shRNA or Lpd-shRNA were plated on collagen supplemented with fibronectin and fixed and stained with a p34 (Arp2/3) antibody. Quantification of the ratio of the length of lamellipodia to length of perimeter of cell. (n=3). Data are represented as mean ± s.e.m. Unpaired t-test; * P ≤ 0.05, **P ≤ 0.0001, NS – not significant. (B) Quantification of protrusion parameter from kymographic analysis of Ctrl-shRNA and Lpd-shRNA2 MDA-MB-231 cells stimulated with EGF. Data are represented as mean ± s.e.m. Unpaired t-test; * P ≤ 0.05, NS – not significant. (C) shRNA-mediated stable knockdown of Lpd in MTLn3 cells was measured by Western blotting. (D) Quantification of mean protrusion speed from kymographic analysis of Ctrl-shRNA and Lpd-shRNA2 MTLn3 cells stimulated with EGF. Data are represented as mean ± s.e.m. Unpaired t-test; NS – not significant. (E) Quantification of protrusion initiation after EGF stimulation in MDA-MB-231 cells stably expressing Ctrl-shRNA or Lpd-shRNA2. Data represented as mean ± s.e.m. Unpaired t-test; * P ≤ 0.05.

Supplemental Figure 4

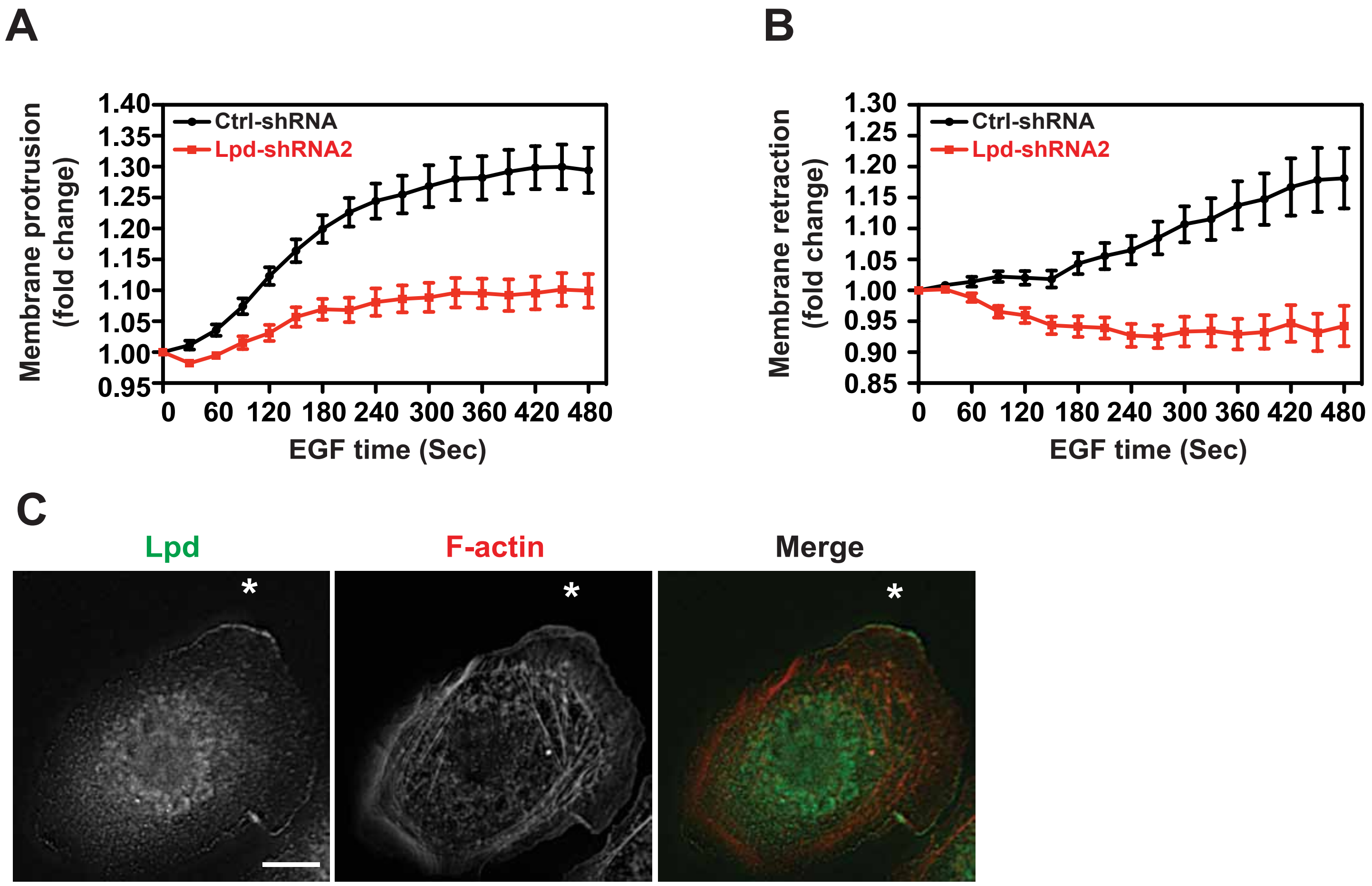

Supplemental Figure 4: Lpd is required for chemosensing.
(A-B) Quantification of membrane protrusion at the front (A) and retraction (B) of Ctrl-shRNA and Lpd-shRNA2 MTLn3 cells versus time after adding EGF. Error bars indicate s.e.m. (C) Immunofluorescence with anti-Lpd (green) and phalloidin (red) of MTLn3 cells stimulated for 1 min with an EGF-filled micropipette (indicated by asterisk). Scale bar, 10 μm.

Supplemental Figure 5

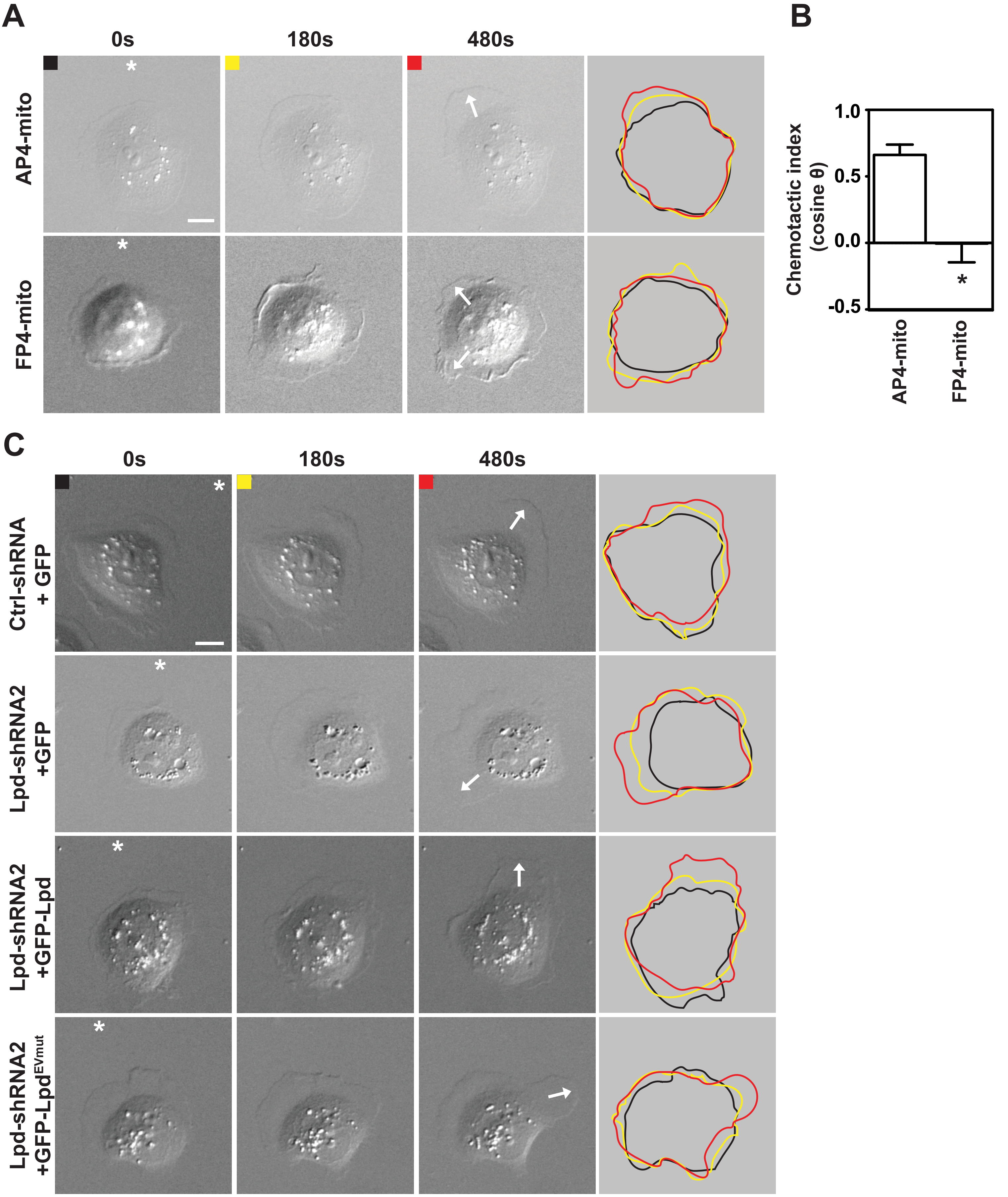

Supplemental Figure 5: Lpd binding to Ena/VASP is required for chemosensing.
(A) Representative micrographs from time-lapse movies of AP4-mito and FP4-mito stably expressed in MTLn3 cells stimulated with an EGF-filled micropipette (asterisk indicates the position of the micropipette). The white arrows on the 480 sec frames indicate the resulting directions of protrusion overtime. Scale bar, 10 µm. Colored lines indicate the contour of the cell. (B) Quantification of the chemotactic index of MTLn3 cells stably expressing AP4-mito or FP4-mito. Over 20 cells analyzed from at least three independent experiments. Data are represented as mean ± s.e.m. Unpaired t-test; * $P \leq 0.05$. (C) Representative micrographs from time-lapse movies of Ctrl-shRNA and Lpd-shRNA2 MTLn3 cells, and transfected with either GFP-vector or GFP-Lpd or GFP-LpdEVmut and stimulated with an EGF-filled micropipette (asterisk indicates the position of the micropipette). The white arrows on the 480 sec frames indicate the resulting directions of protrusion overtime. Scale bar, 10 µm. Colored lines indicate the contour of the cell.

Supplemental Figure 6

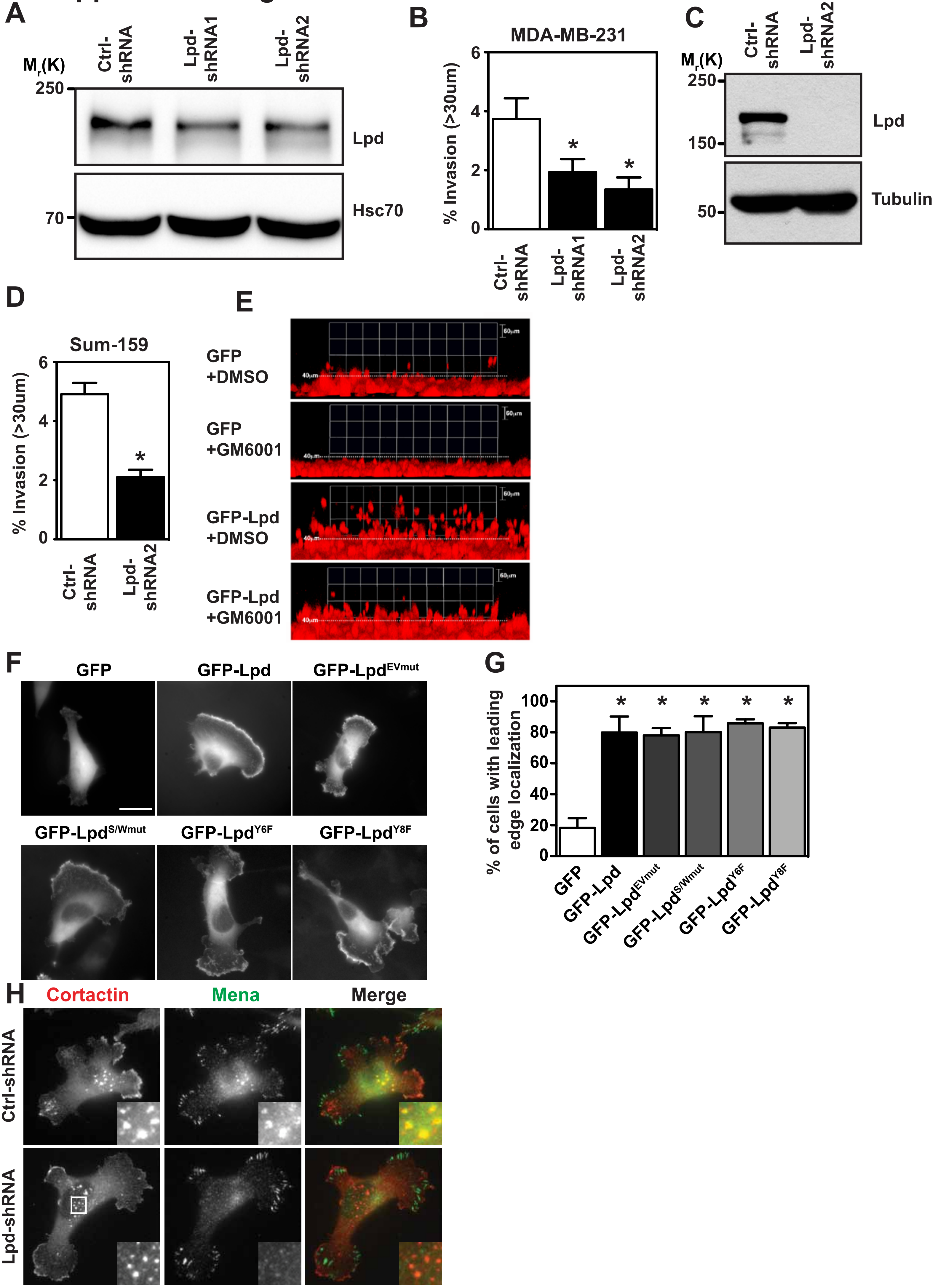

Supplemental Figure 6: Lpd is required for invasion of breast cancer cells.

(A) Western blot analysis of Lpd expression in MDA-MB-231 cells expressing Ctrl-shRNA, Lpd-shRNA1 or Lpd-shRNA2 used in Fig. 5a. Hsc70, loading control. (B) Quantification of 3D-inverted invasion assays using MDA-MB-231 cells expressing Ctrl-shRNA, Lpd-shRNA1 or Lpd-shRNA2 into plugs of collagen I (supplemented with 25µg/ml fibronectin). Invasion is expressed as the proportion of cells that migrate further than 30 µm. Data are represented as mean ± s.e.m; three independent experiments, each performed in duplicate. One-way ANOVA; Dunnett's; * P ≤ 0.05. (C) Western blot analysis of Lpd expression in Sum-159 cells stably expressing Ctrl-shRNA or Lpd-shRNA2. Tubulin, loading control. (D) 3D inverted invasion assay with Sum-159 cells stably expressing Ctrl-shRNA or Lpd-shRNA2 into plugs of collagen I supplemented with 25µg/ml fibronectin. Data are represented as mean ± s.e.m; three independent experiments, each performed in duplicate. Unpaired t-test; * P ≤ 0.05. (E) Inverted invasion assays were performed using MDA-MB-231 breast cancer cells stably expressing mCherry-H2B (labeling the nucleus) transfected with GFP-Lpd or GFP empty vector as control and incubated with 10µM MMP inhibitor GM6001 or DMSO. The nuclei of the cells were visualized using confocal microscopy. (F) Representative images of MDA-MB-231 transfected with GFP-Lpd, GFP-LpdEVmut, GFP-LpdS/Wmut, GFP-LpdY6F, GFP-LpdY8F or GFP empty vector as control. Scale bar, 20 µm. (G) Quantification of percentage of cell displaying Lpd localization to the leading edge. Data are represented as mean ± s.e.m. One-way ANOVA; Dunnett's; * P ≤ 0.05. (H) MDA-MB-231 cells stably expressing Ctrl-shRNA or Lpd-shRNA were plated for 4 hours on 405-gelatin, fixed and immunostained with Mena and Cortactin. White boxes indicate enlarged images shown in insets. Scale bar, 10 µm.

Supplemental Figure 7

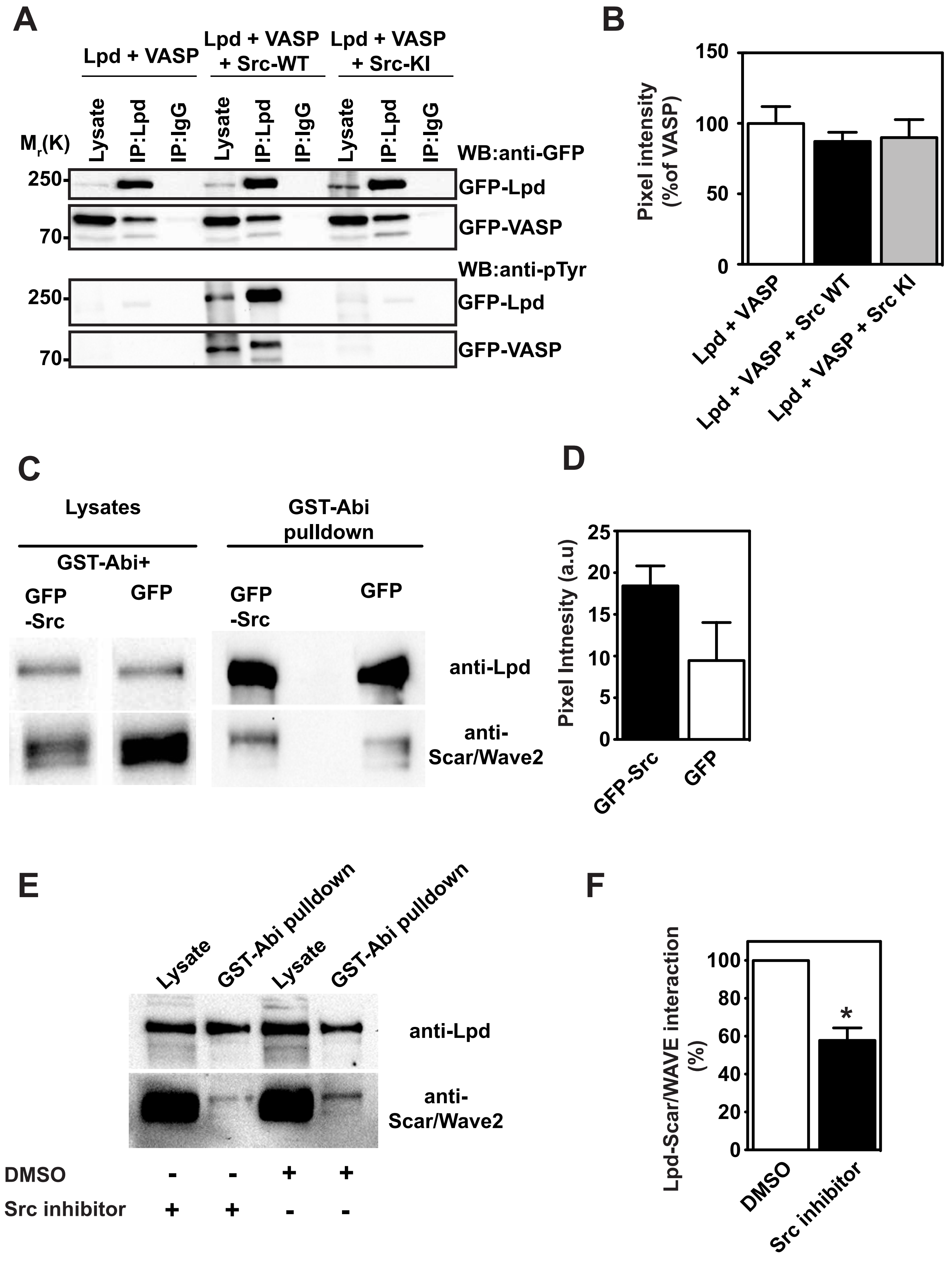

Supplemental Figure 7: Lpd phosphorylation by c-Src does not affect Lpd/VASP interaction.
(A) HEK293FT cells were transfected with GFP-Lpd, GFP-VASP and either Src-WT (wild-type) or Src-KI (kinase-inactive). Immunoprecipitation was performed from cell lysates using Lpd-specific antibody or rabbit IgG as control followed by Western blotting with anti-GFP and anti-phosphotyrosine (pTyr) antibodies. (B) Quantified band intensities of chemiluminescence blots from (A) of GFP-Lpd and GFP-VASP imaged with a CCD camera. VASP was normalised against the immunoprecipitated Lpd. n=3, One-way ANOVA; Dunnett's. Error bars represent s.e.m. (C) HEK293FT cells were transfected with GST-Abi2 (which reduces endogenous Abi and thereby replaces it) and either GFP-Src wild type or GFP as control. GST-Abi2 including the other components of the Scar/WAVE complex and associated proteins were pulled down from cell lysates using Glutathion-beads followed by Western blotting with anti-Lpd, anti-Scar/WAVE2 antibodies to detect endogenous proteins. (D) Quantified band intensities of chemiluminescence blots (C) of Lpd and Scar/WAVE2 imaged with a CCD camera. Scar/WAVE2 was normalized against the pulled down Lpd. n=3, data are represented as mean ± s.e.m. (E) HEK293FT cells were transfected with GST-Abi2 (which reduces endogenous Abi and thereby replaces it) and GFP-Src WT. Cells were incubated for 1 hour with 100µM of the Src inhibitor (KB Src 4) in DMSO or as control with DMSO only. GST-Abi2 including the other components of the Scar/WAVE complex and associated proteins were pulled down from cell lysates using Glutathion-beads followed by Western blotting with anti-Lpd, anti-Scar/WAVE2 antibodies to detect endogenous proteins. (F) Quantified band intensities of chemiluminescence blots (E) of Lpd and Scar/WAVE2 imaged with a CCD camera. Scar/WAVE2 was normalized against the pulled down Lpd. n=3, data are represented as mean ± s.e.m.

Supplemental Figure 8

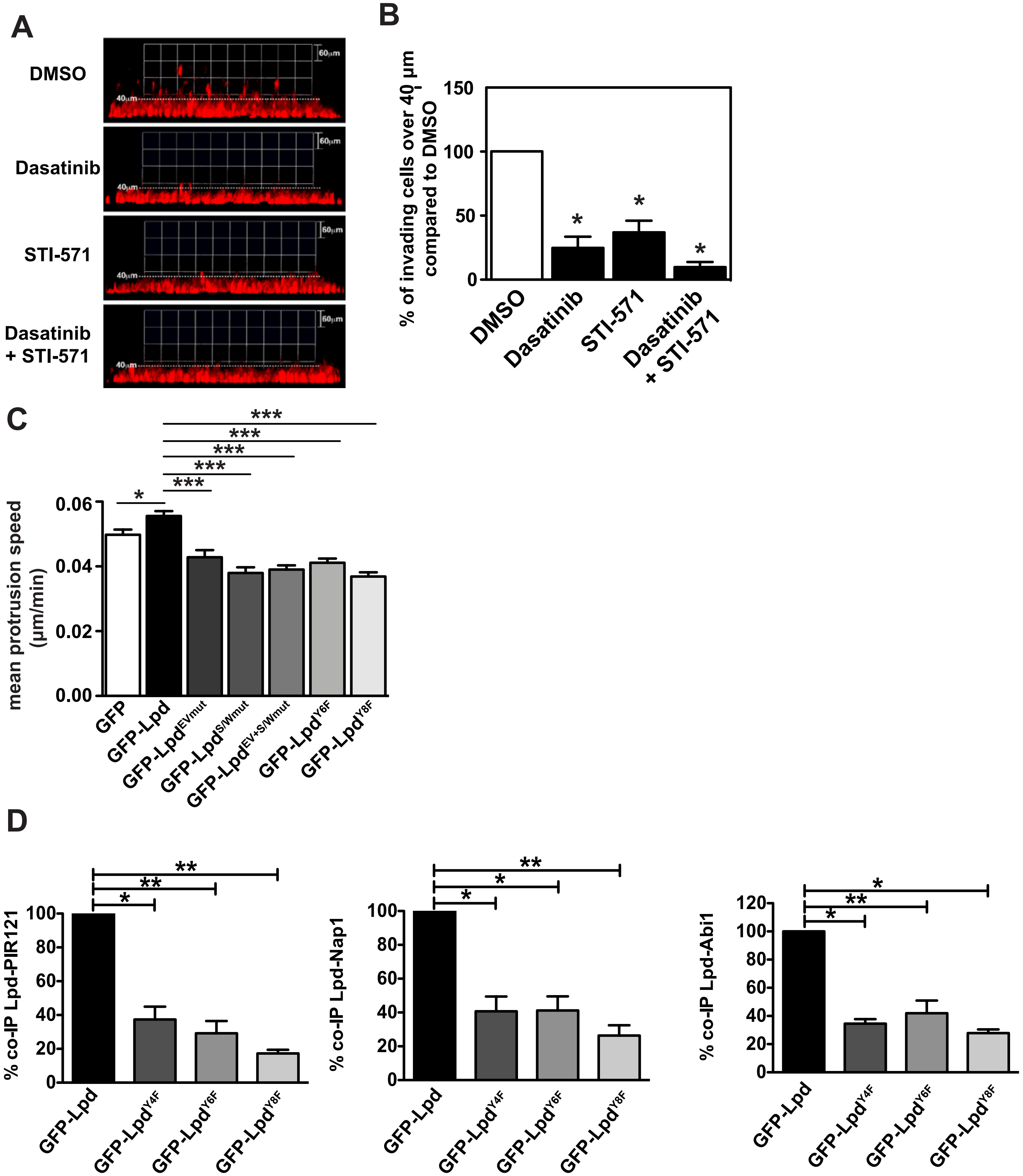

Supplemental Figure 8: Lpd phosphorylation by c-Src is required for its interaction with Scar/WAVE.
(A) Inverted invasion assays were performed using MDA-MB-231 breast cancer cells stably expressing mCherry-H2B (labelling the nucleus). During the three day incubation period of the assay the upper and lower chamber of the transwell was treated with 10nM Dasatinib (a dual c-Src and c-Abl inhibitor, which does not inhibit the EGFR at 10nM (IC50=53nM) (Rix et al., 2007), 10µM STI-571 (c-Abl inhibitor), both inhibitors, or DMSO as control. The nuclei of the cells were visualized using confocal microscopy. (A) The image stacks were processed by Volocity software to make a 3D reconstruction. (B) Quantification of the number of nuclei of invading cells above 40µm using Volocity software. n=4, (with approximately 4000 cells per experiment). Data are represented as mean ± s.e.m., One-way ANOVA; Dunnett's; * P ≤ 0.0001.
(C) Quantification of mean protrusion speed from kymographic analysis of MDA-MB-231 transfected with GFP-Lpd, GFP-LpdEVmut, GFP-LpdS/Wmut, GFP-LpdEV+S/Wmut, GFP-LpdY6F, GFP-LpdY8F or GFP empty vector as control in a 3D environment. n= 35-46 cells for each mutant; from 5 experiments. Data are represented as mean ± s.e.m. One-way ANOVA; Dunnett's; * P ≤ 0.05, *** P< 0.001).
(D) HEK293FT cells were transfected with GFP-Lpd, GFP-Lpd^{Y4F}, GFP-Lpd^{Y6F}, GFP-Lpd^{Y8F} or GFP empty vector as control. Immunoprecipitation was performed from cell lysates using GFP-specific antibody or rabbit IgG as control followed by Western blotting with anti-GFP, anti-Myc. Quantified band intensities of chemilumi-
